# Highly Luminescent Europium(III) Complexes in Solution and PMMA-Doped Films for Bright Red Electroluminescent Devices

**DOI:** 10.3390/molecules28114371

**Published:** 2023-05-26

**Authors:** Zubair Ahmed, Rafael dos Santos Carvalho, Aline Magalhães dos Santos, Francesca Gambassi, Elisa Bandini, Lorenza Marvelli, Lucia Maini, Andrea Barbieri, Marco Cremona

**Affiliations:** 1 Istituto per la Sintesi Organica e la Fotoreattività (ISOF), Consiglio Nazionale delle Ricerche (CNR), Via P. Gobetti 101, 40129 Bologna, Italy; zubairchem011@gmail.com (Z.A.); francesca.gambassi@isof.cnr.it (F.G.); elisa.bandini@isof.cnr.it (E.B.); 2 Departamento de Física, Pontifícia Universidade Católica do Rio de Janeiro (PUC-Rio), Gávea, Rua Marques São Vicente 225, Rio de Janeiro 22451-900, Brazil; rffispuc@yahoo.com.br (R.d.S.C.); aline@aluno.puc-rio.br (A.M.d.S.); 3 Dipartimento di Scienze Chimiche Farmaceutiche ed Agrarie, Università Degli Studi di Ferrara, Via Luigi Borsari 46, 44121 Ferrara, Italy; lorenza.marvelli@unife.it; 4 Dipartimento di Chimica “Giacomo Ciamician”, Università Degli Studi di Bologna, Via Selmi 2, 40126 Bologna, Italy; l.maini@unibo.it

**Keywords:** europium complexes, crystal structure, photoluminescence, electroluminescence, OLED

## Abstract

This paper reports the synthesis, structure, photophysical, and optoelectronic properties of five eight-coordinate Europium(III) ternary complexes, namely, [Eu(hth)_3_(L)_2_], bearing 4,4,5,5,6,6,6-heptafluoro-1-(2-thienyl)-1,3-hexanedione (hth) as a sensitizer and L = H_2_O (**1**), dpso (diphenyl sulphoxide, **2**), dpsoCH_3_ (4,4′-dimethyl diphenyl sulfoxide, **3**), dpsoCl (bis(4-chlorophenyl)sulphoxide, **4**), and tppo (triphenylphosphine oxide, **5**) as co-ligands. The NMR and the crystal structure analysis confirmed the eight-coordinate structures of the complexes in solution and in a solid state. Upon UV-excitation on the absorption band of the β-diketonate ligand hth, all complexes showed the characteristic bright red luminescence of the Europium ion. The tppo derivative (**5**) displayed the highest quantum yield (up to 66%). As a result, an organic light-emitting device, OLED, was fabricated with a multi-layered structure—ITO/MoO_3_/mCP/SF3PO:[complex **5**] (10%)/TPBi:[complex **5**] (10%)/TmPyPB/LiF/Al—using complex **5** as the emitting component.

## 1. Introduction

In recent years, much work has been conducted regarding the design and development of visible-light-emitting lanthanide complexes, which have found applications in different fields, such as biological imaging [1], sensoring [2,3,4], solar energy conversion [5], and optoelectronics [6]. The lanthanide complexes, particularly those with the Eu(III) ion, still attract huge amounts of attention due to their ability to provide very intense red light with narrow emission bands and long emission lifetimes (up to ms). However, the f–f transitions arising from the Eu ion are Laporte-forbidden transitions; consequently, they require strong light-absorbing chromophores to achieve their intense red emission at 612–620 nm, corresponding to a ^5^D_0_ → ^7^F_2_ transition. This is an electric dipole hypersensitive transition and its intensity and shape are very sensitive to the changes in the coordination structures constructed by the organic chromophores [7].

The β-diketones ligands are widely investigated and known to sensitize the emission of lanthanide ions very effectively since they show a very strong π,π* absorption band in the UV region with suitable excited state energy [8,9]. Generally, the Eu(III) ion in combination with β-diketone ligands in a 1:3 molar ratio constructs an aquo-complex [Eu(β-diketone)_3_(H_2_O)_2_]. The two coordinated water molecules may be replaced either by a fourth diketonate anion or by mono-/polydentate co-ligands that may be functionalized so as to provide suitable light-harvesting and a subsequent energy transfer onto the Eu(III) ion [8,9,10]. With a judicious choice of co-ligands, the photophysical properties of the resulting ternary Eu complex can be effectively tuned. Additionally, they can protect the complex from the detrimental effect of high-frequency oscillators, such as C–H, N–H and O–H, present in the vicinity of the lanthanide ion, which quenches the emission [11]. Another important key to boosting the overall luminescence efficiency of the lanthanide complexes is the asymmetry in the inner coordination sphere, which is related to the transition probability of the emitting state, controlled by relaxing the selection rules [12,13].

The introduction of the co-ligands into the lanthanide complex, in particular, the O-donor monodentate ligands bearing two/three phenyl groups, improves the photophysical properties (emission quantum yield and lifetimes) of the ternary complexes [14,15,16,17,18]. Among the monodentate auxiliary ligands, triphenyl phosphine oxide (tppo) coordinates with the lanthanide ion, improving the photophysical properties of the complexes significantly [14,15,16,17,18,19]. Moreover, the presence of this ligand in a complex improves the electroluminescence performance, charge transport characteristics, and light-emitting properties of the lanthanide-complex-based devices as well [20,21]. Additionally, such complexes show good thin-film-forming properties, such as high volatility, light-weight feel, good transparency, and easy thermal evaporation—basic requirements for developing efficient and stable OLEDs. These are the main reason why the lanthanide β-diketonate complexes are more and more frequently used in optoelectronic applications. Recently, various visible OLEDs based on lanthanide complexes have been reportedly designed. However, efficient and stable red-light-emitting OLEDs based on Eu(III) complexes are few. There is a strong demand for the development of such devices since they find potential applications in biomedical imaging, which in turn will enrich the lighting industries.

This paper reports on five ternary Eu(III) complexes, with the general formulas of [Eu(hth)_3_(L)_2_], with 4,4,5,5,6,6,6-heptafluoro-1-(2-thienyl)-1,3-hexanedione (hth) and L = H_2_O (**1**), dpso (diphenyl sulphoxide, **2**), dpsoCH_3_ (4,4′-dimethyl diphenyl sulfoxide, **3**), dpsoCl (bis(4-chlorophenyl)sulphoxide, **4**), and tppo (triphenylphosphine oxide, **5**). The NMR and crystal structure analysis confirm the eight-coordinate structures of the complexes in solution and in a solid state. The photophysical properties of the complexes, in solution and in doped plastic films, are discussed and presented. Complex **5** was found to be highly luminescent and stable and was used as an emitting component in the fabrication of an OLED with the multilayered structure ITO/MoO_3_/mCP/SF3PO:[complex **5**] (10%)/TPBi:[complex **5**] (10%)/TmPyPB/LiF/Al.

## 2. Results and Discussion

### 2.1. Synthesis

**Scheme 1:**
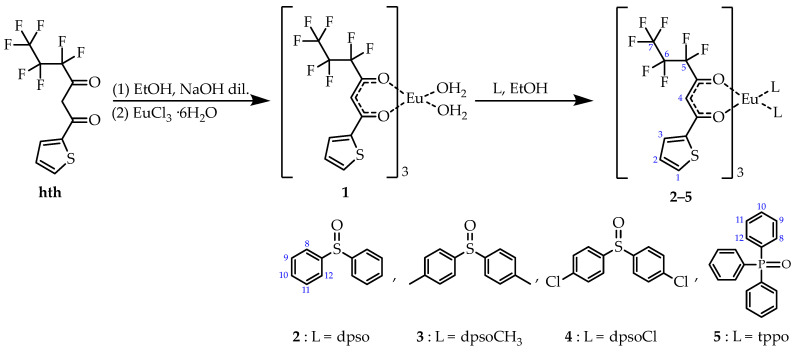
Synthetic route of the complexes.

Complexes **2**–**5** were obtained in two steps; the first was the formation of Europium aquo-complex **1**, achieved by mixing a basic ethanolic solution of 4,4,5,5,6,6,6-heptafluoro-1-(2-thienyl)-1,3-hexanedione (hth) (3 mmol) with EuCl_3_·6H_2_O (1 mmol) dissolved in water. The pale yellow precipitate of complex **1** was then isolated and subsequently treated with a co-ligand L (L = dpso, dpsoCH_3_, dpsoCl, and tppo) at a 2:1 L/Eu ratio to obtain the relevant complexes (**2**–**5**) in good yield (Scheme 1, Section 3).

### 2.2. NMR Analysis

A NMR characterization of complexes **1**–**5** was performed by means of the ^1^H, ^13^C, ^19^F, and HSQC spectra, as reported in the Supplementary Materials. The presence of a paramagnetic nucleus, such as Europium, resulted in down-shifted and/or up-shifted and broadened signals with respect to the free ligands.

The ^1^H NMR spectra of complexes **1**–**5** (Figure S1a–f) could be compared by dividing the spectral zone into three parts: one above 7.5 ppm, where the aromatic protons of the ancillary ligand are located; a zone between 7.5 and 5.0 ppm, for the thiophene protons; and a zone between 4.0 and 1.0 ppm, for the methine proton of the β-diketone ligand (H4) and the CH_3_ protons in dpsoCH_3_. Since complexes **1**–**5** shared the same β-diketone ligand but differed in terms of the co-ligand (H_2_O or L), the ratio of each component could be determined by using the integrals of the proton spectra. The Eu:hth:L ratio found was 1:3:2, which confirmed the stoichiometric ratio used in the synthetic procedure.

The ^19^F spectra (Figure S2a–f) showed the three distinct signals of the CF_2_CF_2_CF_3_ chain (hth ligand) with integrals corresponding to the F-number, demonstrating that the three bidentate ligands were arranged symmetrically around the Europium atom and that no other fluorinated species or isomers were present. Compared to the free hth ligand, the paramagnetic shift of the Eu(III) in complexes **1**–**5** mainly affected the CF_2_ in position 5, due to its proximity to the coordination center (from −121.6 to −126.7 ppm), and the CF_2_ in position 6 with less evidence (form −127.0 to −128.9 ppm); meanwhile, CF_3_ was not significantly shifted. In addition, these signals are broadened and less resolved confirming that coordination has occurred.

**Figure 1:**
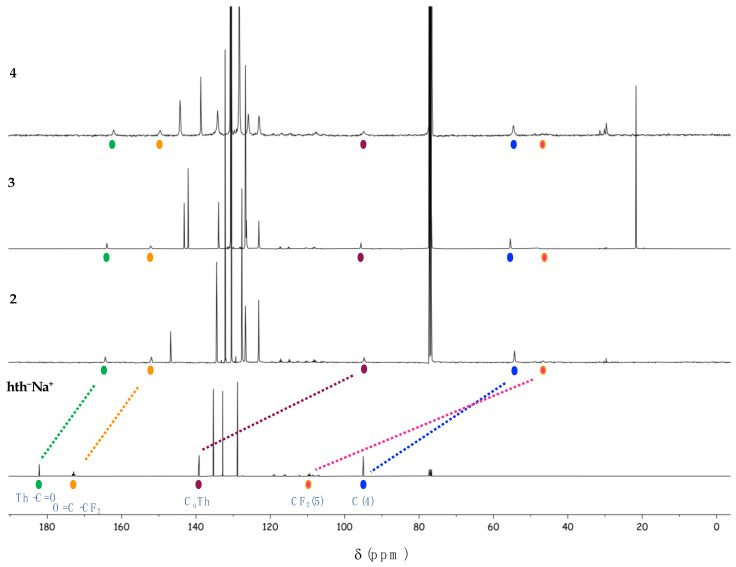
^13^C NMR spectra of the **hth–Na^+^** ligand and complexes **2**–**4** in CDCl_3_.

The ^13^C NMR spectra (Figure S3a–d) of complexes with fluorinated ligands were rather difficult to acquire and interpret for several reasons: (i) C–F spin-spin interactions caused the lines to repeatedly split; (ii) the presence of quaternary carbons, as in the CF_2_CF_2_CF_3_ chain of the hth ligand, substantially decreased the intensity; (iii) a signal-broadening effect occurred due to the presence of the paramagnetic europium ion; and finally, (iv) there was a high molecular weight. Despite the difficulties reported, we were able to obtain good results for complexes **2**–**4** and, by comparison with the free ligand, significant information was obtained. All peaks varied in their positions, but the nuclei closest to the coordination center underwent the greatest shifts. In particular there were significant changes in the peaks of the carbonyls (ca. 20 ppm upfielded from 182 and 173 ppm), the carbon in position 4 (from 95 ppm in the free hth to about 50–55 ppm in the complexes), the thiophene quaternary carbon (from 139 to 95 ppm), and the CF_2_ in position 5 (from 110 ppm to 47 ppm), as reported in Figure 1.

### 2.3. Single Crystal X-ray Crystallography

**Figure 2:**
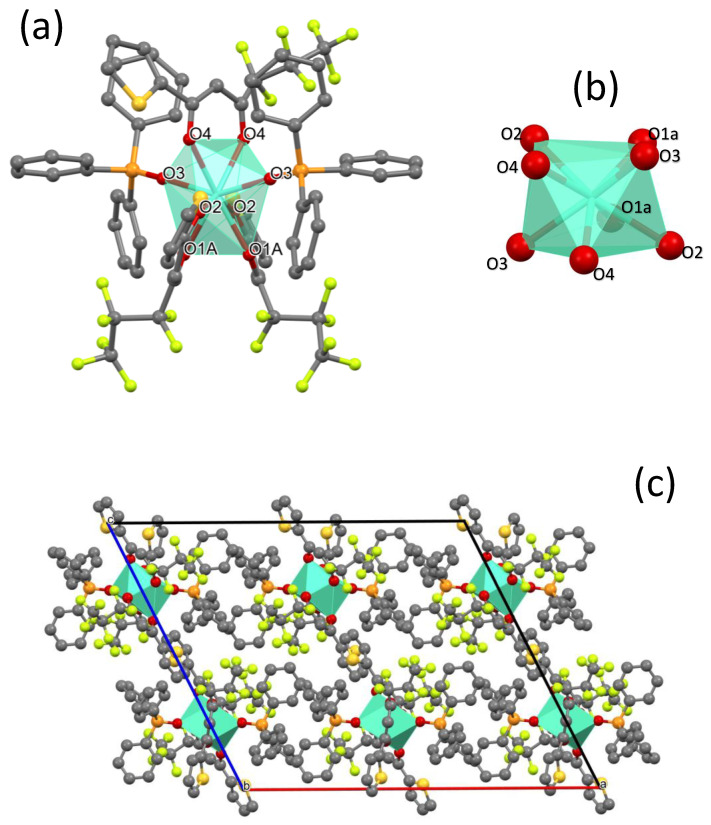
(**a**) Molecular structure of complex **5**. The top hth is on the 2-fold axis and only one orientation is shown. For the hth molecules on the bottom, only one disordered position is shown for the sake of clarity. (**b**) The polyhedron of the complex **5**. (**c**) Packing view along the b-axis. Hydrogen atoms are omitted and only one disordered position is shown for the sake of clarity.

The single crystal of complex **5**, obtained from hexane solution, was solved by single crystal X-ray diffraction (XRD). The details are given in Tables S1 and S2 (Supplementary Materials). The [Eu(hth)_3_(tppo)_2_] (**5**) is a neutral mononuclear species that crystallizes in the C2/c space group of the monoclinic system. The Eu atom lies on the 2-fold axis; hence, the asymmetric unit consists of one half [Eu(hth)_3_(tppo)_2_] of the complex (Figure 2a). Since the molecule and the ligands did not possess a 2-fold axis, the symmetry was allowed by lying the acid carbon of one hth on the 2-fold axis, with the thiophene ring and the alkyl chain disordered over two positions. The disorder also affected the integer hth ligand and the acid carbon; one coordinating oxygen atom and the fluorinated chain were disordered over two positions refined with occupancies of 0.52 and 0.48. The complex is an eight-coordinate system, where the Euion is attached to six O-atoms of three hth ligands and two O-atoms of two tppo co-ligands, with the EuO_8_ coordination polyhedron having a C_2_ symmetry (Figure 2b). The crystal packing showed that the Eu ions were separated from each other by a minimum distance of 12.25 Å (Figure 2c), which is sufficient for minimizing the energy migrations between them. This phenomenon helps in improving the luminescence efficiency by preventing non-radiative energy losses. The tppo units in the complex were not co-axial since the bond angles (O–Eu–O) between the Eu ion and tppo ligands were 152.4° from each other. The average length of an Eu–O(hth) bonds is 2.401 (Table S2, Supplementary Materials), which was in good agreement with the reported structures with hth: [Eu_2_(hth)_6_Bpm] (2.302 Å) [22] and [Eu(hth)_3_phen] (2.373 Å) [23]. In contrast, the length of the Eu–O(tppo) bond was 2.350(4) Å and it agreed well with the average distance (2.33(3) Å) observed in Eu complexes with tppo, which were collected at room temperature [24]. In the crystal structure of complex **5**, the molecules interacted with each other mainly through weak intermolecular C–H/F⋯F–C interactions.

### 2.4. Photophysical Study

**Figure 3:**
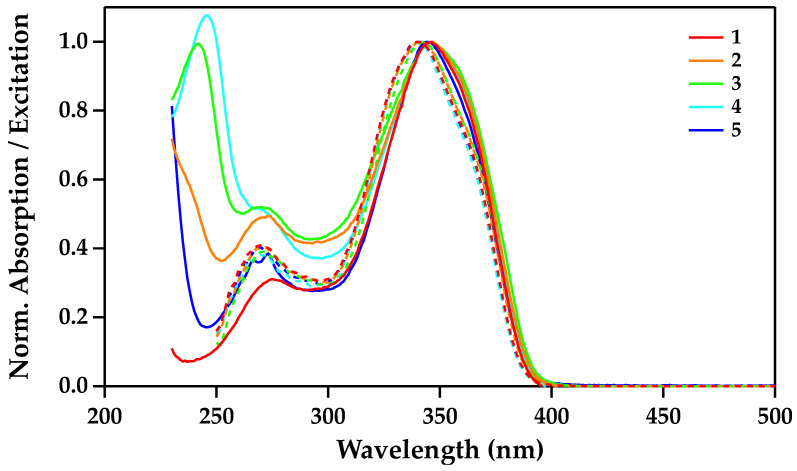
Normalized absorption (full line) and excitation (dotted line) spectra of the complexes in dichloromethane solution at rt.

The absorption spectra of complexes **1**–**5** recorded in dichloromethane at room temperature (rt) are reported in Figure 3 and the relevant main photophysical parameters are presented in Table 1. The spectra showed a strong absorption band in the UV region between 225 and 400 nm which can be ascribed to π,π* transitions, with the maximum peaking at about 345 nm and a molar absorption coefficient (ε) on the order of 6 × 10^4^ M^−1^·cm^−1^. The absorption spectra of the complexes were comparable to the sum of the three hth and two co-ligands, thus confirming the proposed composition.

The excitation spectra of the complexes were recorded in a dichloromethane solution at rt, monitoring the ^5^D_0_ → ^7^F_2_ f-f transition of the Eu(III) ion at 612 nm. The spectra showed a broad band, peaking around 345 nm and covering the 300–400 nm region (Figure 3). The spectra almost mimicked their corresponding absorption spectra, suggesting that the emission of Eu(III) ion was induced by the π,π* absorption of the ligands.

**Figure 4:**
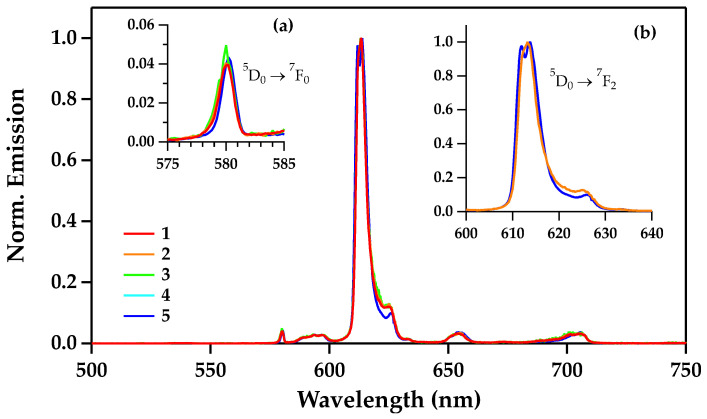
Normalized emission spectra of the complexes in dichloromethane solution at rt. Magnifications of (**a**) the ^5^D_0_ → ^7^F_0_, and (**b**) the ^5^D_0_ → ^7^F_2_ transitions.

The emission spectra of complexes **1**–**5** were recorded in dichloromethane, acetonitrile solutions and PMMA films at rt with an excitation wavelength of 345 nm (Figure 4 and Figure S6a,b). The main photophysical parameters are presented in Table 1. The emission spectra of all the complexes showed a bright red luminescence due to the characteristic transitions (^5^D_0_ → ^7^F*_J_*_=0–4_) of the Eu(III) ion. The single peak of the transition ^5^D_0_ → ^7^F_0_ at 580 nm suggested a stable structure (no dissociation of any coordinated organic ligands) of the complexes in both the solution and the PMMA film since the changes in the band shape of this transition reflected the number of different emitting sites in the complex (Figure 4, inset). The transition ^5^D_0_ → ^7^F_1_ was a magnetic dipole transition and and it is not affected by changes in the environment around the metal ion around the metal ion [25]. On the other end, the hypersensitive electric dipole transition ^5^D_0_ → ^7^F_2_ at 612 nm was very sensitive to the chemical environments (ligand/solvent) around the metal ion [26]. The very intense ^5^D_0_ → ^7^F_2_ transition with respect to the ^5^D_0_ → ^7^F_1_ transition suggested that the complexes did not possess a center of inversion [15]. Moreover, the band shape of the ^5^D_0_ → ^7^F_2_ transition was almost identical for complexes **2**–**4** (having dpso/its derivatives as co-ligands), while being different for complex **5** (with tppo as co-ligands)**,** which displayed a split peak, indicating the influence of the co-ligands on this hypersensitive transition.

The PMMA-doped films of the complexes showed a high quantum yield as compared to their corresponding dichloromethane solution (Table 1). Indeed, the dichloromethane solvent had oscillator modes that could quench the excited states of the Eu ion, therefore decreasing the luminescence efficiency by internal conversion [11]. In contrast, the rigid environment of the PMMA matrix hindered the non-radiative processes, thus allowing a higher quantum efficiency. In contrast, the complexes in the acetonitrile solution showed the highest quantum efficiency—almost double that of the PMMA-doped films. It was assumed that the acetonitrile with a high dielectric constant value (37.5), as compared to dichloromethane (4.81) and PMMA (3.9), could polarize the complexes more effectively, thus changing the symmetry of the ligand field around the Eu ion and providing the low symmetry to the complexes; this lead to a higher quantum efficiency [15]. The hypersensitive ^5^D_0_ → ^7^F_2_ transition clearly showed different band shapes in different media, suggesting the presence of different environments around the Eu ion (Figure S7).

The photoluminescence quantum yield (*ϕ*) of complex **5** was 66% which was the highest among the complexes studied in this paper (Table 1). It was also higher than those reported for [Eu(tmh)_3_(diphenyl(p-tolyl)phosphine oxide)] (0.6%) [27], [Eu(hfaa)_3_(bpyO2)] (35% solution and 40% solid) [28], [Eu-(NTA)_3_bpy] (51%) [29], and [Eu(hfaa)_3_(DPPPO)] (61%) [13]; it was comparable to those reported for [Eu(tta)_2_(terpyridine-carboxylate)] (66% solid; 62% PMMA and 60% PVA) [30], (tmh = 2,2,6,6-tetramethylheptane-3,5-dione, hfaa = hexafluoroacetylacetone, tta = 2-thenoyltrifluoroacetylacetonate, bpyO2 = 2,2′-bipyridine-N,N′-dioxide, NTA = 1-(2-naphthoyl)-3,3,3-trifluoroacetonate, and DPPPO = {bis[o-(diphenylphosphoryl)phenyl]phenylphosphane).

The luminescence lifetime curves of the complexes in the dichloromethane, acetonitrile and PMMA-doped films, recorded at room temperature with an excitation of 345 nm, all displayed mono-exponential behavior. This suggested that the complexes contained only one Eu-emitting site, both in solutions and in the films. The lifetime values observed in acetonitrile were almost two-fold of those observed for the dichloromethane and solid films. In complex **1**, longer emission lifetimes could be related to the replacement of the coordinated water molecules by the acetonitrile molecules. In complexes **2**–**5**, the reason could be attributed to the low symmetry of the complexes in acetonitrile solution, which could improve the radiative constant values and lead to longer emission lifetimes. Among all of the complexes, complex **5** showed the longest emission lifetime of 597 μs, which was higher than those reported for [Eu(btfa)_3_phen] (210 μs) [31] and [Eu(tmh)_3_(diphenyl(p-tolyl) phosphine oxide)] (500 μs) [27], and comparable to those reported for [Eu(tmh)_3_(diphenyl(p-tolyl)phosphine oxide)] (601 μs) [27] and [Eu(NTA)_3_L] [29] (620/662 μs) (L = bipyridine/phenenthroline).

The dpso, dpsoCH_3_, dpsoCl, and tppo co-ligands could all effectively sensitize the ^5^D_0_ excited state level of the Europium ion (Figure S8, Supplementary Materials). The relevant sensitization efficiencies are presented in Table S3 (Supplementary Materials). Efficiencies close to 100% were calculated for all complexes.

### 2.5. Electroluminescence Properties

**Figure 5:**
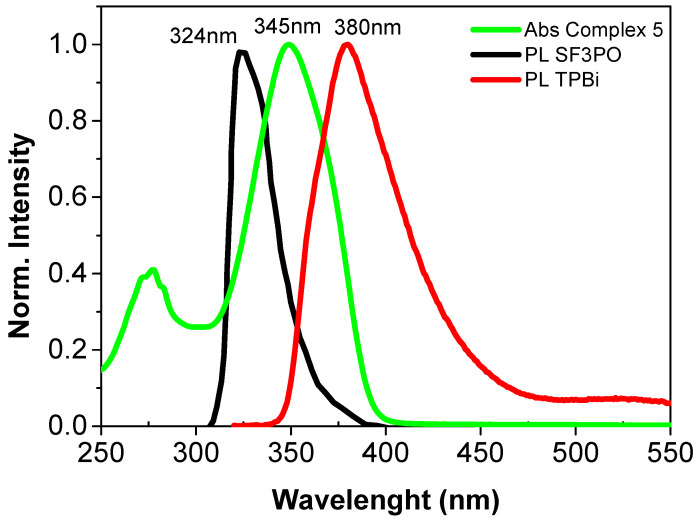
Photoluminescence spectra of the organic matrixes and the absorption spectrum of the complex **5**.

**Figure 6:**
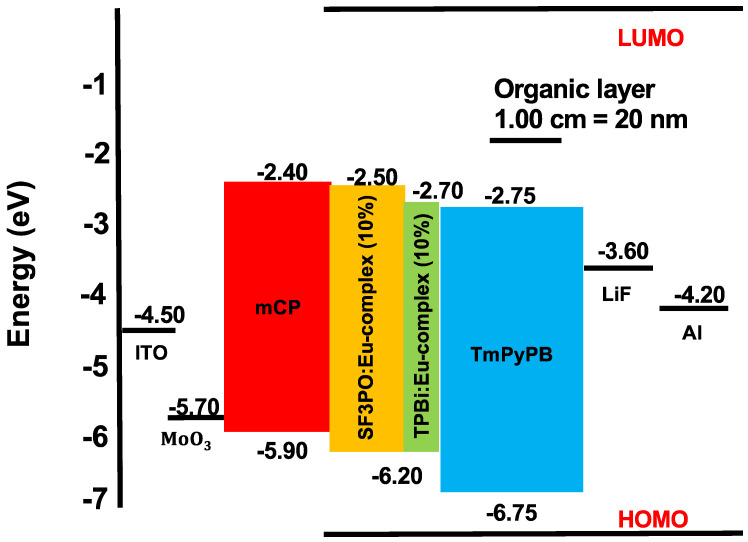
Energy level diagram of the OLED based on complex **5**.

A multilayered device with the structure ITO/MoO_3_/mCP/SF3PO:[complex **5**] (10%)/TPBi:[complex **5**] (10%)/TmPyPB/LiF/Al was fabricated, as inspired by the literature [32,33]. To confirm if a sufficient amount of energy would transfer from organic matrixes to the Eu complex, the complex was doped into SF3PO and TPBi matrixes with a percent ratio of 90:10 (matrixes:complex **5**, *w*/*w*). The absorption spectrum of complex **5** overlapped well with the emission spectra of the matrixes, suggesting an efficient energy transfer from the matrixes to the complex (Figure 5). Another key element for designing an optimum device was matching the energies of the triplet states of all of the organic layers; it was also necessary to check if they had good abilities to inject/transfer the electrons/holes in the device [16,17,18]. The HOMO and LUMO energy levels were determined as −5.82 and −2.71 eV, respectively, and were a good match with the HOMO/LUMO of the SF3PO (−6.20 and −2.50 eV) and TPBi (−6.20 and −2.70 eV) organic layers. It was assumed that this result would lead to an efficient device. The energy level diagram of the OLED containing the HOMO/LUMO levels and the triplet states of the materials used is shown in Figure 6.

**Figure 7:**
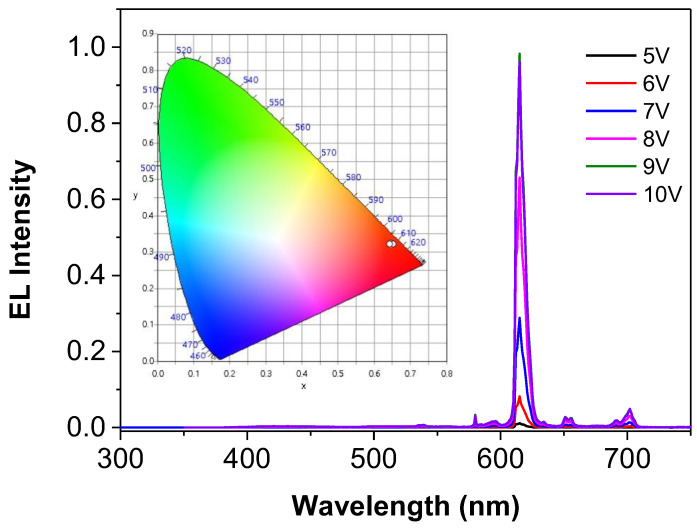
Electroluminescence spectra and CIE coordinates of the OLED based on complex **5**.

**Figure 8:**
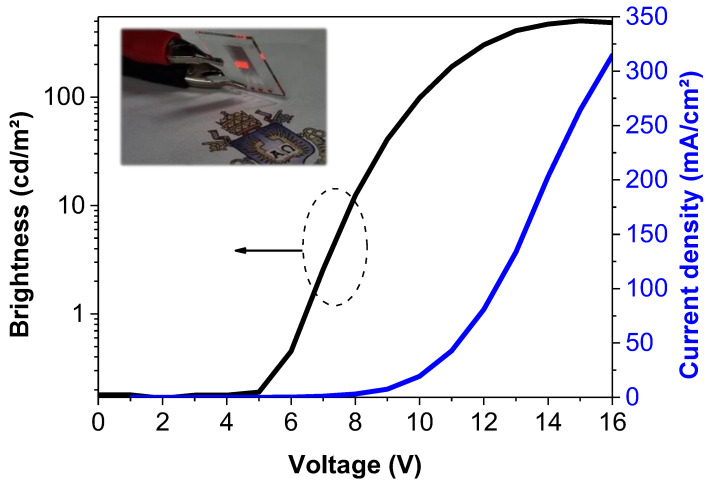
Current density—Voltage (J-V) and Brightness—Voltage (I-V) curves and the image of the OLED on.

The device based on complex **5** had a turn voltage of 5.0 V. The EL spectrum of the device showed the characteristic transitions of the Eu(III) ion at 533, 576, 589, 611, 647, and 698 nm corresponding to ^5^D_1_ → ^7^F_1_, ^5^D_0_ → ^7^F_0_, ^5^D_0_ → ^7^F_1_, ^5^D_0_ → ^7^F_2_, ^5^D_0_ → ^7^F_3_ and ^5^D_0_ → ^7^F_4_, respectively. The CIE coordinates (0.6440–0.6537, 0.3216–0.3245) were calculated using the measured electroluminescence spectrum and the software LED ColorCalculator v7.77 (from OSRAM SYLVANIA) for 5 V, 8 V, and 10 V applied voltage, showing quite a pure Eu emission color (Figure 7). The measurements were obtained at 1 μA–3 mA driving currents, which lead to all transitions of the Eu ion in the electroluminescence spectrum. Moreover, the FWHM of ^5^D_0_ → ^7^F_2_ transition at 612 nm was 8 nm. The electroluminescence spectrum was almost similar to that of the photoluminescence spectrum of complex **5**, except for a very weak visible emission at 400 nm that could be from the NPB matrix, and a relatively strong ^5^D_1_ → ^7^F_1_ transition. It could be ascribed to the strong quenching of the singlet excitons produced on the matrices by the complex, a possibility owing to the efficient energy transfer from organic matrices to the complex (tppo) (via Forster transfer). The devices displayed a maximum brightness of 504.6 cd/m^2^ with a current density (J) of 314 mA/cm^2^ at 16 V (Figure 8). The External Quantum Efficiency (EQE) was calculated using the methodology described by Cusumano et al. [34]. The EQE maximum was 1.05% at 9 V with 0.22 mA. The device was stable until 20 V. The stability and the performance of the OLED could be related to the presence of tppo co-ligand in complex **5**, which made a thermally stable complex and possessed good electron-transport properties. Moreover, the electrical performance of the present device was comparable to that of a device based on the closely related complex [Eu(tta)_3_(phen)] (maximum brightness, 505 cd/m^2^ obtained at 12 V) [35], lower than that of [Eu(DBM)_3_(bath)] (brightness, 820 cd/m^2^ at 3 V) [36], but had better color purity than [Eu(btfa)_3_Py-im] and [Eu(DBM)_3_phen] [32,33].

## 3. Materials and Methods

The compounds EuCl_3_·6H_2_O (99.9%, Aldrich) and 4,4,5,5,6,6,6-heptafluoro-1-(2-thienyl)-1,3-hexanedione (hth) (Apollo Scientific), and the co-ligands dpso (97%, Aldrich), dpsoCH_3_ (97%, Aldrich), dpsoCl (97%, Aldrich), and tppo (>98%, Fluka) were used without further purification. The NMR spectra of the complexes, in CDCl_3_ (99.8%, Cambridge Isotope Labs) at 25 °C, were recorded on a Varian Mercury 400 MHz and on an Agilent DD2 500 MHz equipped with the OneNMR probe NMR spectrometers. The infrared spectra were recorded using a VERTEX 70v Bruker FT-IR spectrometer, examining powder samples in anhydrous KBr via diffuse reflection spectroscopy. Elemental analyses were carried out using a Thermo Scientific™ FLASH 2000 CHNS/O Analyzer at the Department of Chemical, Pharmaceutical and Agricultural Sciences, University of Ferrara.

### 3.1. Synthesis of Complexes

The 4,4,5,5,6,6,6-heptafluoro-1-(2-thienyl)-1,3-hexanedione (0.5 g, 1.55 mmol) was dissolved in 10 mL ethanol and 10 mL of an aqueous solution of sodium hydroxide (0.062 g, 1.55 mmol) was added. The mixture was heated to 60 °C and stirred for half an hour. Thereafter, Eu(III) chloride hexahydrate (0.19 g, 0.52 mmol) dissolved in 15 mL of water was added to the reaction mixture and a pale-yellow precipitate was immediately formed. After 30 min the precipitate was filtered, washed with water, and dried under a vacuum. Aquo-complex **1** was obtained in 63% yield. Complexes **2**–**5** were prepared by adding 0.1 mmol of the co-ligand (dpso = 20 mg; dpsoCH_3_ = 23 mg; dpsoCl = 26 mg; and tppo = 28 mg) to an ethanolic solution of complex **1** (58 mg, 0.05 mmol) and stirring this mixture for 5 h. Subsequently, the mixture was kept for 5 days until the solvent evaporated, leaving a pale-yellow precipitate. The complexes were then purified by crystallization with dichloromethane and hexane.

hth^–^Na^+^, ^1^H NMR (400 MHz, CDCl_3_), (δ) ppm: 7.83 (dd, 1H, *J*_1_ = 1.16 Hz, *J*_2_ = 3.90 Hz, H1), 7.75 (dd, 1H, *J*_1_ = 1.16 Hz, *J*_2_ = 4.96 Hz, H3), 7.18 (dd, 1H, *J*_1_ = 4.96 Hz, *J*_2_ = 3.90 Hz, H2), 6.48 (s, 1H, H4). ^19^F NMR (376 MHz, CDCl_3_), (δ) ppm: −80.88 (t, 3F, *J* = 9.1 Hz, CF_3_), −121.60 (m, 2F, CF_2_(5)), −127.03 (m, 2F, CF_2_(6)). ^13^C NMR (100 MHz, CDCl_3_), (δ) ppm: 182.23 (C=O), 172.97 (t, *J*_C–F_ = 26.56 Hz, C=O), 139.18 (C_q_Th), 135.38 (C1), 132.85 (C3), 128.87 (C2), 119.33–106.20 (m, CF_2_CF_2_CF3), 95.06 (m, C4).

[Eu(hth)_3_(H_2_O)_2_] **1**, yield (63%, 0.38 g). ^1^H NMR (400 MHz, CDCl_3_), (δ) ppm: 29.98 (bs, 4H, H_2_O), 7.14 (bs, 3H, H1), 6.53 (bs, 3H, H2), 6.26 (bs, 3H, H3), 1.71 (bs, 3H, H4). ^19^F NMR (470 MHz, CDCl_3_), (δ) ppm: −81.57 (bs, 3F, CF_3_), −126.81 (bs, 2F, CF_2_(5)), −128.92 (bs, 2F, CF_2_(6)). FT-IR (ν, cm^−1^): 3107, 1610, 1535, 1505, 1457, 1413, 1347, 1293, 1281, 1225, 1117, 1094, 1083, 1063, 1017. Elemental analysis: Anal. Calcd. for C_30_H_16_EuF_21_O_8_S_3_: C, 31.29; H, 1.40 S, 8.35%. Found: C, 31.65; H, 1.76; S, 9.96%.

[Eu(hth)_3_(dpso)_2_] **2**, yield (88%, 67 mg). ^1^H NMR (500 MHz, CDCl_3_), (δ) ppm: 11.10 (bs, 8H, H8, H12), 8.06 (m, 8H, H9, H11), 7.96 (t, 4H, H10), 6.79 (bs, 3H, H1), 6.33 (bs, 3H, H2), 5.64 (bs, 3H, H3), 1.69 (bs, 3H, H4). ^19^F NMR (470 MHz, CDCl_3_), (δ) ppm: −81.51 (bt, 3F, CF_3_), −126.40 (bs, 2F, CF_2_(5)), −128.86 (bs, 2F, CF_2_(6)). ^13^C NMR (125 MHz, CDCl_3_), (δ) ppm: 164.39 (C=O), 152.00 (bt, C=O), 146.80 (C–S), 134.43 (C1), 132.16 (C10) 130.47 (C9, C11), 127.64 (C8, C12), 126.69 (C3), 123.13 (C2), 119.76–112,34 (qt, *J*_1_ = 286 Hz, *J*_2_ = 33 Hz, CF_3_), 110.68–105.60 (tq, *J*_1_ = 264 Hz, *J*_2_ = 35 Hz, CF_2_ (6)), 94.76 (bt, C_q_Th), 54.33 (C4), 49.47–43.86 (m, CF_2_(5)). FT-IR (ν, cm^−1^): 3066, 1706, 1610, 1531, 1515, 1499, 1446, 1414, 1346, 1281, 1230, 1117, 1090, 1063, 1016.

[Eu(hth)_3_(dpsoCH_3_)_2_] **3**, yield (63%, 50 mg). ^1^H NMR (400 MHz, CDCl_3_), (δ) ppm: 10.97 (bs, 8H, H8, H12), 7.80 (bs, 8H, H9, H11), 6.72 (bs, 3H, H1), 6.29 (bs, 3H, H2), 5.57 (bs, 3H, H3), 2.62 (s, 12H), 1.87 (bs, 3H, H4). ^19^F NMR (376 MHz, CDCl_3_), (δ) ppm: −81.40 (bt, 3F, CF_3_), −125.89 (bs, 2F, CF_2_(5)), −128.69 (bs, 2F, CF_2_(6)). ^13^C NMR (125 MHz, CDCl_3_), (δ) ppm: 163.97 (C=O), 152.20 (bt, C=O), 143.17 (C–S), 142.11 (C–CH_3_), 133.91 (C1), 130.65 (C9, C11), 126.68 (C8, C12), 126.45 (C3), 123.09 (C2), 119,80–112.30 (qt, *J*_1_ = 285 Hz, *J*_2_ = 33 Hz, CF_3_), 110.80–105.63 (tq, *J*_1_ = 263 Hz, *J*_2_ = 36 Hz, CF_2_ (6)), 95.63 (C_q_Th), 55.45 (C4), 49.81–45.83 (m, CF_2_(5)), 21.66 (CH_3_). FT-IR data (ν, cm^−1^): 3104, 3059, 1706, 1610, 1531, 1514, 1496, 1466, 1414, 1346, 1281, 1230, 1117, 1090, 1060, 1009.

[Eu(hth)_3_(dpsoCl)_2_] **4**, yield (96%, 78 mg). ^1^H NMR (400 MHz, CDCl_3_), (δ) ppm: 12.66 (bs, 8H, H8, H12), 8.35 (bs, 8H, H9, H11), 6.70 (bs, 3H, H1), 6.18 (bs, 3H, H2) 5.16 (bs, 3H, H3), 1.20 (bs, 3H, H4). ^19^F NMR (470 MHz, CDCl_3_), (δ) ppm: −81.61 (bs, 3F, CF_3_), −126.55 (bs, 2F, CF_2_(5)), −129.01 (bs, 2F, CF_2_(6)). ^13^C NMR (125 MHz, CDCl_3_), (δ) ppm: 162.17 (C=O), 149.69 (C=O), 144.29 (C–S), 138.72 (C–Cl), 134.17 (C1), 130.69 (C9, C11), 128.39 (C8, C12), 125.94 (C3), 123.04 (C2), 119.40–105.50 (m, CF_2_(6), CF_3_), 94.88 (C_q_Th), 54.60 (C4), 49.0–43.0 (m, CF_2_(5)). FT-IR data (ν, cm^−1^): 3088, 1708, 1609, 1530, 1514, 1499, 1476, 1414, 1346, 1281, 1230, 1117, 1090, 1082, 1059, 1011.

[Eu(hth)_3_(tppo)_2_] **5**, yield (96%, 81 mg). ^1^H NMR (400 MHz, CDCl_3_), (δ) ppm: 7.75 (m, 15H, H8-12), 6.67 (bs, 3H, H1), 6.33 (bs, 3H, H2), 5.87 (bs, 3H, H3), 3.18 (bs, 3H, H4). ^19^F NMR (470 MHz, CDCl_3_), (δ) ppm: −81.14 (bs, 3F, CF_3_), −125.02 (bs, 2F, CF_2_(5)), −128.04 (bs, 2F, CF_2_(6)). FT-IR data (ν, cm^−1^): 3063, 1612, 1531, 1512, 1495, 1474, 1438, 1417, 1347, 1278, 1227, 1178, 1122, 1116, 1097, 1085, 1064, 1017. Elemental analysis: Anal. Calcd. for C_66_H_42_EuF_21_O_8_P_2_S_3_: C, 47.41; H, 2.53; S, 5.75%. Found: C, 47.71; H, 2.89; S, 5.98%.

### 3.2. XRD Crystallography

The slow evaporation of the hexane solutions of complex **5** [Eu(hth)_3_(tppo)_2_] led to single crystals of X-ray crystallographic quality. The crystal data of complex **5** were collected on an Oxford Xcalibur instrument with MoKα radiation (λ = 0.71073 Å) and graphite monochromator at room temperature. The SHELXT program [37] was used for structure solution and refinement based on F^2^. Non-hydrogen atoms were refined anisotropically. The Eu atom and the acid carbon of one hth ligand laid on the 2-fold axis; the occupancy of the atoms of this hth ligand was constrained to a 0.5 occupancy. The other hth ligand, which was in the general position, was characterized by the acid carbon atom, one coordinating oxygen atom, and the fluorinated chain disordered over two positions that were refined with occupancies of 0.52 and 0.48. Hydrogen atoms were added in calculated positions. The crystallographic data are summarized in Table S1 (Supplementary Materials). The Mercury software [38] was used for graphical representations. The CCDC 2010956 contains the supplementary crystallographic data for this paper. These data can be obtained free of charge from the Cambridge Crystallographic Data Centre via www.ccdc.cam.ac.uk/data_request/cif (accessed on 23 May 2023).

### 3.3. Photophysics

All solvents used for the photophysical studies were of spectroscopic grade (Uvasol^®^ Merck) and were used without further purification. Square optical Suprasil Quartz (Hellma) cuvettes of a 1 cm path length were used for the absorption and emission measurements at room temperature. The absorption spectra of the dilute solutions were obtained by using a Perkin Elmer Lambda 950 UV/VIS/NIR spectrophotometer. The molar absorption coefficients (ε) were calculated by applying the Lambert–Beer law to low absorbance spectra (*A* < 1) recorded at successive dilutions. The steady-state photoluminescence spectra were measured in the right angle mode using an Edinburgh FLS920 fluorimeter equipped with a Xenon arc lamp and a Hamamatsu R928P Peltier-cooled photomultiplier tube. The concentration of the sample solutions was adjusted to obtain the absorption values of A < 0.1 at the excitation wavelength. All emission spectra were corrected for the wavelength-dependent phototube response between 200 and 900 nm using a calibration curve provided by the manufacturer. The luminescence quantum yields in the solution were evaluated by comparing the wavelength-integrated intensities of the corrected spectra, with reference to the [Ru(bpy)_3_]Cl_2_ (*ϕ_r_* = 0.040 in air-equilibrated H_2_O) standard (Carlo Erba) [39]. The luminescence lifetimes were obtained using a TCSPC apparatus (HORIBA) equipped with a TBX Picosecond photon-detection module and NanoLED/SpectraLED pulsed excitation sources. The analysis of luminescence decay profiles against time was accomplished using the Decay Analysis Software DAS v6.5 (HORIBA).

### 3.4. Device Fabrication

The OLED was assembled using heterojunctions containing a multilayer structure, with 2 nm of MoO_3_ (99.99%, LumTec) as the injection layer and 30 nm of mCP (99.5%, LumTec) (1,3-Bis(N-carbazolyl)benzene) as a hole-transporting layer. Complex **5** worked as an emitting layer (10% *w*/*w*) in 20 nm of SF3PO (99%, LumTec) and as an emitting layer (10% *w*/*w*) in 10 nm of TPBi (99.5%, LumTec). As an electron-transporting layer, 40 nm of TmPyPB (99.8%, LumTec) (1,3,5-Tris(3-pyridyl-3-phenyl)benzene) were evaporated. Finally, a 100 nm thick aluminum (99.99%, Kurt and Lesker) film was deposited as a cathode onto 0.1 nm of LiF (99.99%, LumTec). All organic semiconductor materials were used with no purification process. The different layers of the device were sequentially deposited in a high-vacuum environment via thermal evaporation onto ITO substrates with a sheet resistance of 15 Ω/square. The ITO substrate was initially cleaned by ultrasonication, using a detergent solution followed by toluene degreasing and then cleaning again via ultrasonication with pure isopropyl alcohol. Finally, the substrate was dried using nitrogen gas. The base pressure was 6.6 × 10^−5^ Pa; whereas, during the evaporation process, the pressure was ~10^−4^ Pa. The deposition rates for organic compounds ranged from 0.3 Å/s to 0.8 Å/s. The layer thickness was controlled in situ through a quartz crystal monitor and confirmed with a profilometer measurement. The fabricated device had an active area of around 3 mm^2^ and operated in a forward bias voltage with ITO as the positive electrode and Al as the negative one. The current-luminance-voltage properties were measured by using a Keithley source measurement unit (Keithley 2400) and Newport Powermeter Model 1936-C.

## 4. Conclusions

Five eight-coordinate Eu(III) ternary complexes, with the general formula [Eu(hth)_3_(L)_2_], were synthesized by two-step reactions. The NMR and crystal structure analysis confirm the eight-coordinate structures of the complexes in solution and in a solid state. The complexes showed improved photophysical properties (quantum efficiency and emission lifetime) in acetonitrile, especially when compared to those in the dichloromethane solution and PMMA-doped films. It is concluded that the high dielectric constant and coordinative nature of acetonitrile leads to the complexes being in low symmetry, which results in improved photophysical properties. Among all of the complexes, complex **5** was found to be the most stable and efficient. Consequently, it was used as an emitting component in the fabrication of OLED with the multilayered structure ITO/MoO_3_/mCP/SF3PO:[complex **5**] (10%)/TPBi:[complex **5**] (10%)/TmPyPB/LiF/Al.

## Figures and Tables

**Table 1:** Photophysical properties of Eu complexes **1**–**5** in solution and solid state.

	*λ*_max_, nm	ε, M^−1^·cm^−1^	*ϕ*, %			*τ*, μs		
	DCM		DCM	ACN	PMMA	DCM	ACN	PMMA
**1**	346	58,800	17.4	55.7	23	275	574	303
**2**	346	64,300	12.4	59.4	26	255	584	318
**3**	346	54,800	8.93	51.3	26	227	576	325
**4**	346	60,000	12.4	57.5	24	229	583	309
**5**	344	64,500	54.2	65.8	56	308	597	470

## Data Availability

Not applicable.

## Supplementary Materials

The following supporting information can be downloaded at: https://www.mdpi.com/article/10.3390/molecules28114371/s1, Figure S1: ^1^H-NMR spectra of the free ligand hth and the complexes **1**–**5** in CDCl_3_; Figure S2: ^19^F-NMR spectra of the free ligand hth and the complexes **1**–**5** in CDCl_3_; Figure S3: ^13^C-NMR spectra of the free ligand hth and the complexes **2**–**4** in CDCl_3_; Figure S4: HSQC NMR spectra of the complexes **2**–**3** in *d*-CHCl_3_; Figure S5: Vibrational spectra of the complexes **1**–**5**; Figure S6: Normalized emission spectra of the complexes **1**–**5** in acetonitrile solution and PMMA film at rt; Figure S7: Band shapes of the ^5^D_0_ → ^7^F_2_ transition of complex **2** in CH_2_Cl_2_, CH_3_CN solutions and PMMA film; Figure S8: Energy levels diagram. The data for the 5Dn excited states of Eu(III) are from ref [40], the triplet level of the bdiketonate ligand hth is from ref [41], and the triplet levels of co-ligands dpso, dpsoCH3, dpsoCl are from ref [42] and tppo from ref [43]. Table S1: Crystal data and structure refinement of the complex **5**, the calculations were performed on the disordered [Eu(hth)_3_(tppo)_2_] which contains the O1A and O1B; Table S2: Selected bond lengths (Å) of the complex **5**; Table S3: Sensitization efficiencies. References [40,41,42,43] are citied in the Supplementary Materials.
