# Supplementary material for: Highly Luminescent Europium(III) Complexes in Solution and PMMA-Doped Films for Bright Red Electroluminescent Devices
Source: Molecules. 2023 May 26;28(11):4371. doi: 10.3390/molecules28114371 (PMC10254153; doi:10.3390/molecules28114371)
Supplement: Supplementary file 1 [file molecules-28-04371-s001.zip › molecules-2399659-supplementary.pdf]

molecules

Supporting Information

Highly Luminescent Europium(III) Complexes in Solution and PMMA-Doped Films for Bright Red Electroluminescent Devices

Zubair Ahmed [1], Rafael dos Santos Carvalho [2], Aline Magalhães dos Santos [2], Francesca Gambassi [1], Elisa Bandini [1], Lorenza Marvelli [3], Lucia Maini [4], Andrea Barbieri [1,*] and Marco Cremona [2,*]

[1] Istituto per la Sintesi Organica e la Fotoreattività (ISOF), Consiglio Nazionale delle Ricerche (CNR), Via P. Gobetti 101, 40129 Bologna, Italy
[2] Departamento de Física, Pontifícia Universidade Católica do Rio de Janeiro (PUC-Rio), Gávea, Rua Marques São Vicente 225, Rio de Janeiro 22451-900, Brazil
[3] Dipartimento di Scienze Chimiche Farmaceutiche ed Agrarie, Università Degli Studi di Ferrara, Via Luigi Borsari 46, 44121 Ferrara, Italy
[4] Dipartimento di Chimica "Giacomo Ciamician", Università Degli Studi di Bologna, Via Selmi 2, 40126 Bologna, Italy
* Correspondence: andrea.barbieri@isof.cnr.it (A.B.); cremona@fis.puc-rio.br (M.C.)

Figure S1a-f. ^{1}H-NMR spectra of the ligand **hth^{-}Na^{+}** and the complexes **1-5** in $CDCl_3$ 2-7

Figure S2a-f. ^{19}F-NMR spectra of the ligand **hth^{-}Na^{+}** and the complexes **1-5** in $CDCl_3$ 8-13

Figure S3a-d. ^{13}C-NMR spectra of the ligand **hth^{-}Na^{+}** and the complexes **2-4** in $CDCl_3$.................... 14-17

Figure S4a-b. HSQC NMR spectra of the complexes **2-3** in $CDCl_3$ 18-19

Figure S5a-e. Vibrational spectra of the complexes **1-5** 20-24

Figure S6a-b. Normalized emission spectra of the complexes **1-5** in acetonitrile solution and PMMA film at rt...... 25-26

Figure S7. Band shapes of the $^{5}D_0 \rightarrow ^{7}F_2$ transition of complex **2** in CH_2Cl_2, CH_3CN solutions and PMMA film 27

Figure S8. Energy levels diagram....................28

Table S1. Crystal data and structure refinement of the complex **5** 29

Table S2. Selected bond lengths of the complex **5**....................29

Table S3. Sensitization efficiencies 30

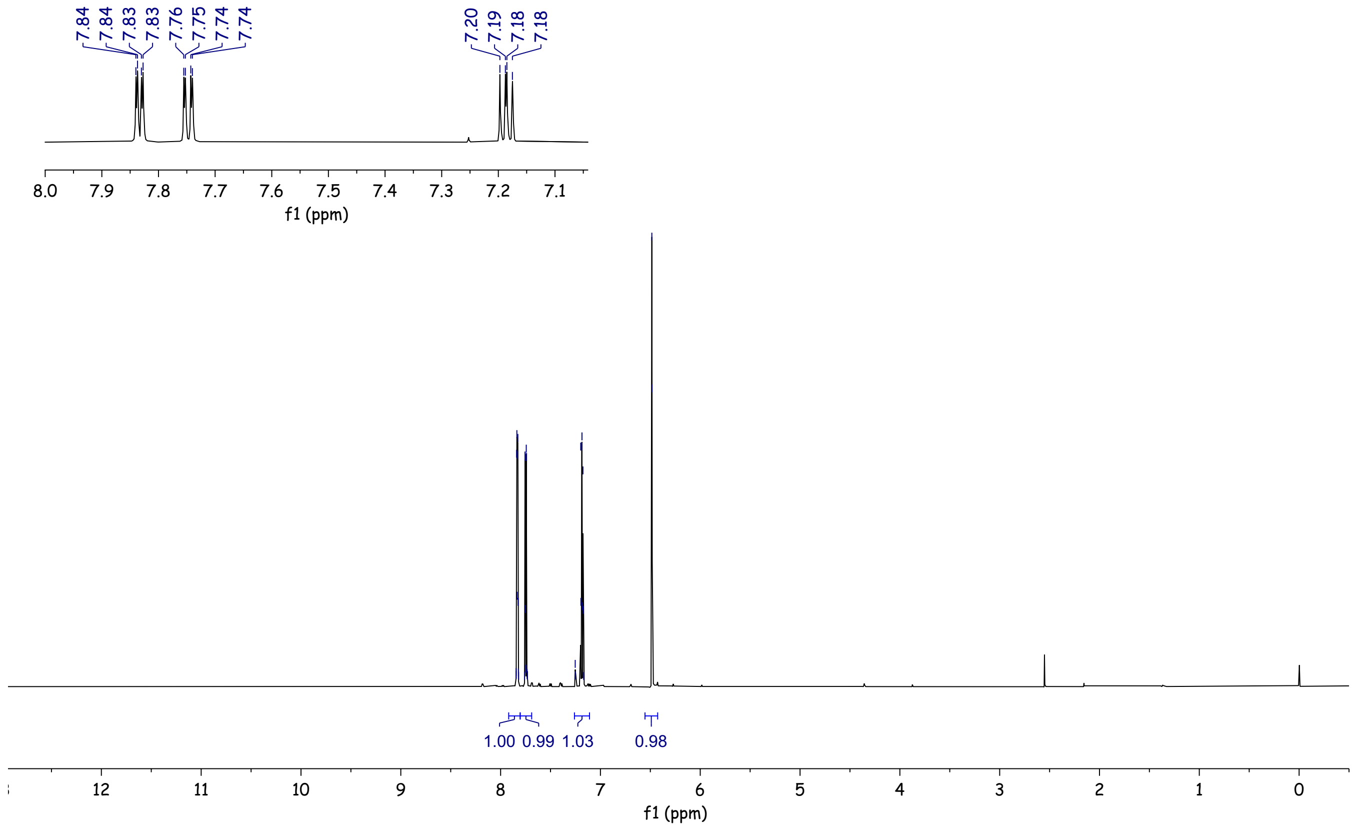

Figure S1a. ^{1}H-NMR spectra of the ligand **hth^{-}Na^{+}** in $CDCl_3$.

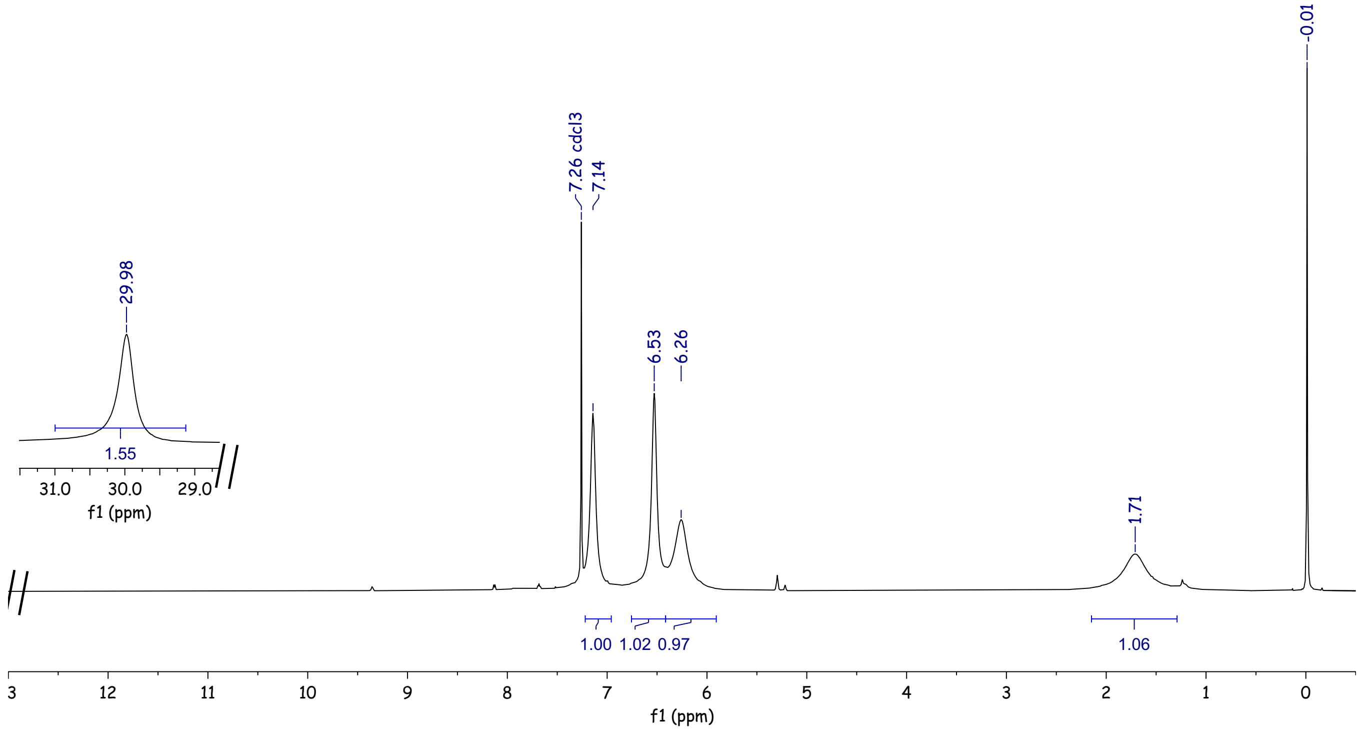

Figure S1b. ^{1}H-NMR spectra of the complex [Eu(hth)$_3$(H$_2$O)$_2$] (**1**) in $CDCl_3$.

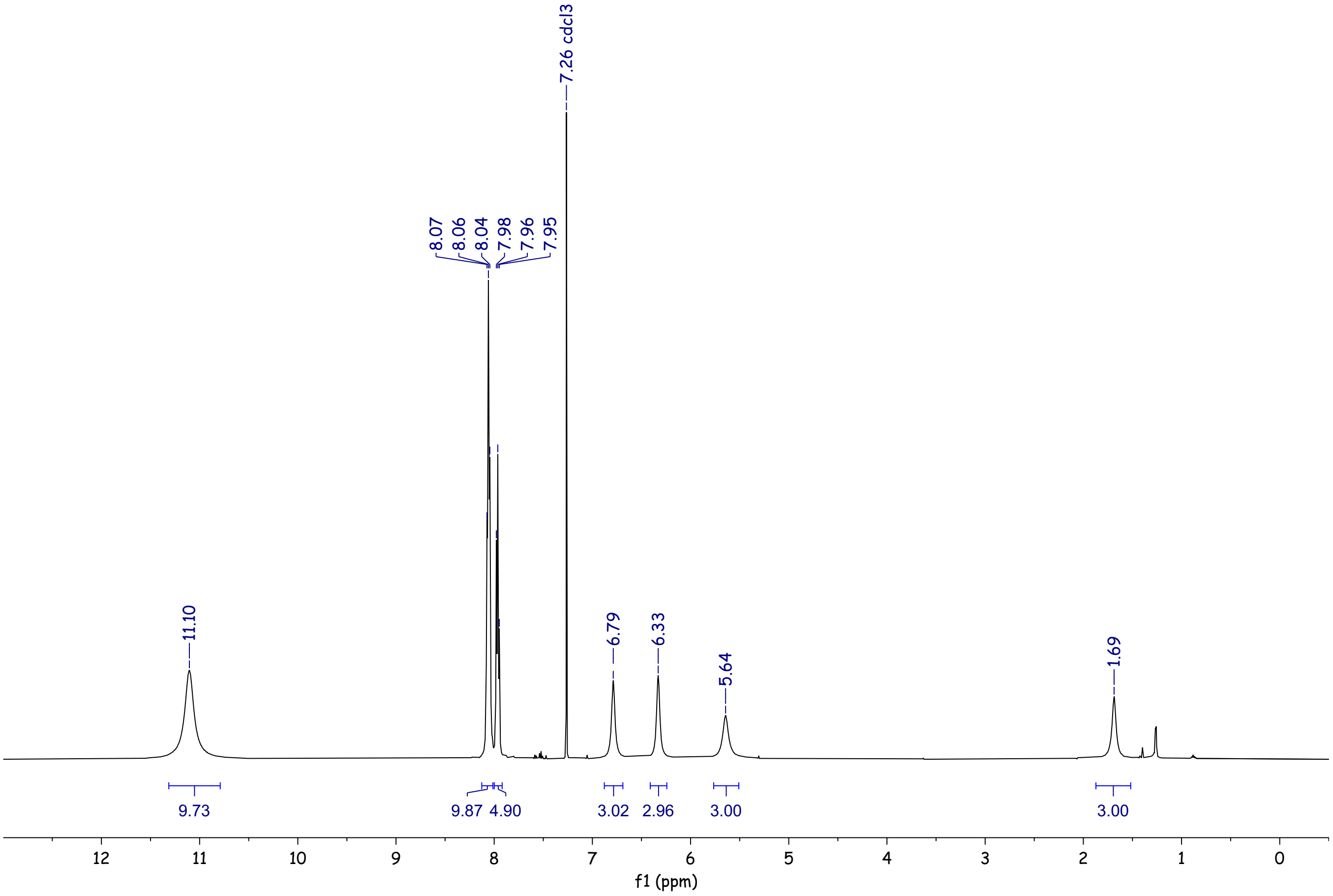

Figure S1c. ^{1}H-NMR spectra of the complex [Eu(hth)$_3$(dpso)$_2$] (**2**) in $CDCl_3$.

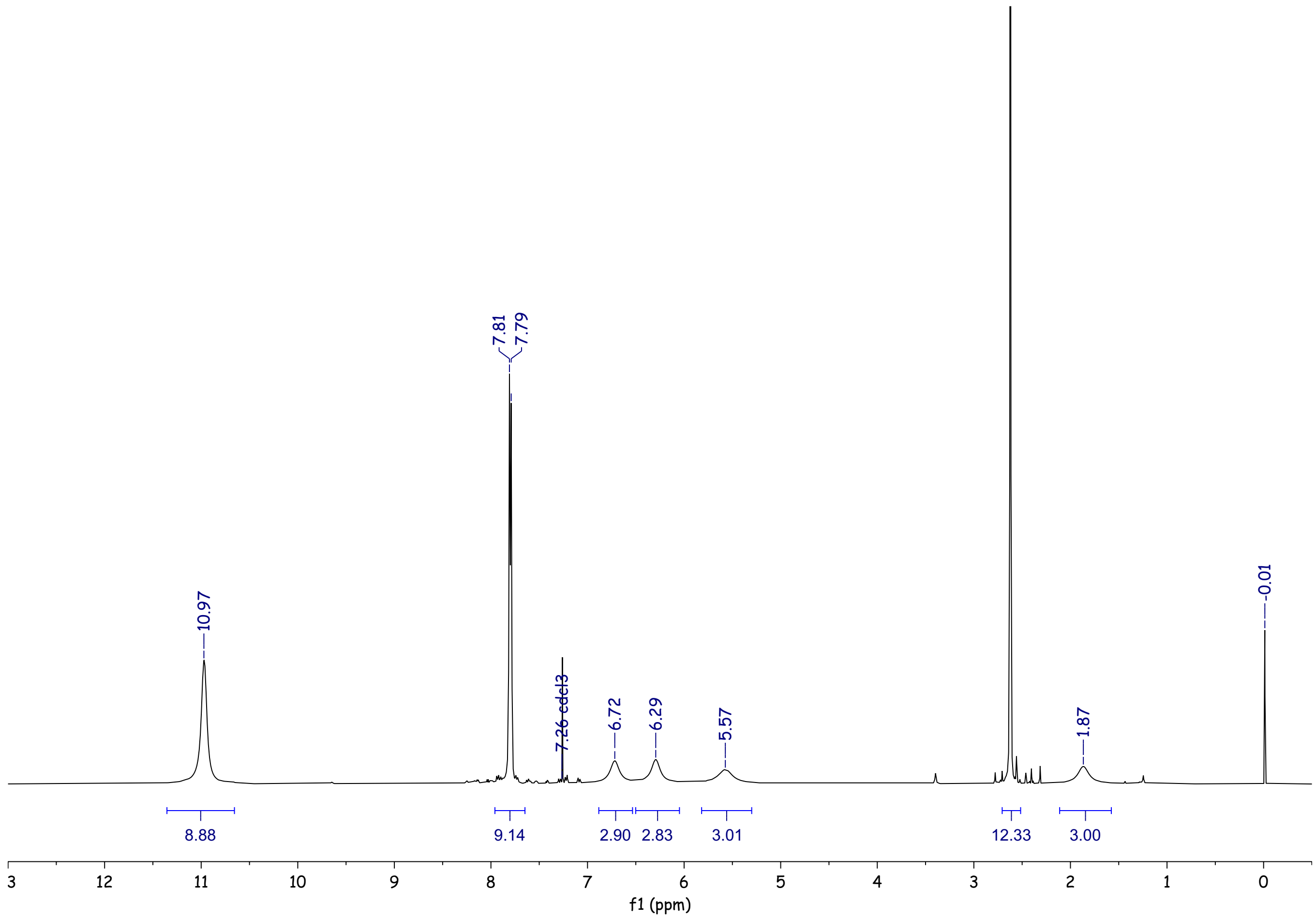

Figure S1d. ^{1}H-NMR spectra of the complex [Eu(hth)$_3$(dpsoCH$_3$)$_2$] (**3**) in $CDCl_3$.

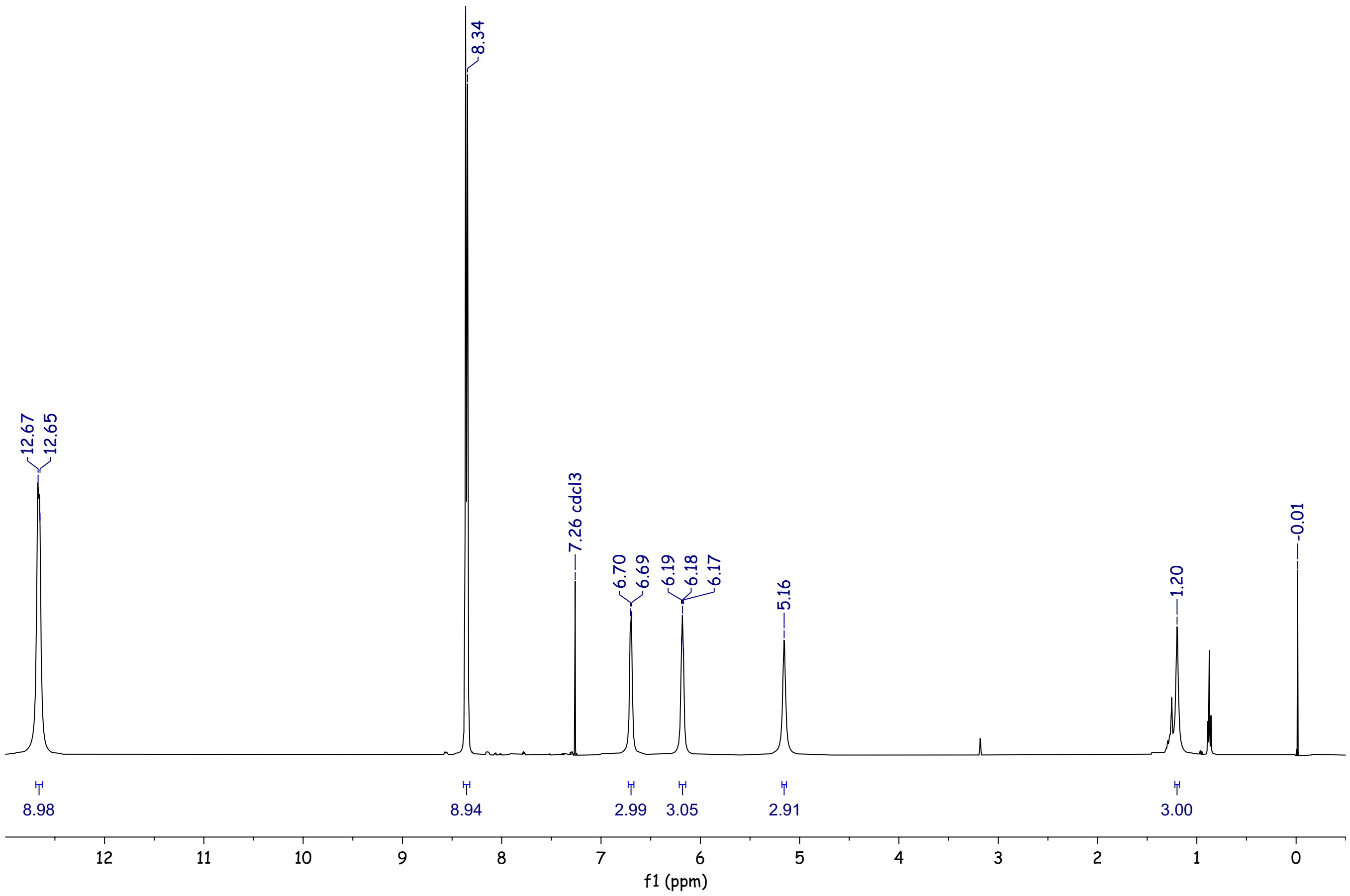

Figure S1e. ^{1}H-NMR spectra of the complex [Eu(hth)$_3$(dpsoCl)$_2$] (**4**) in $CDCl_3$.

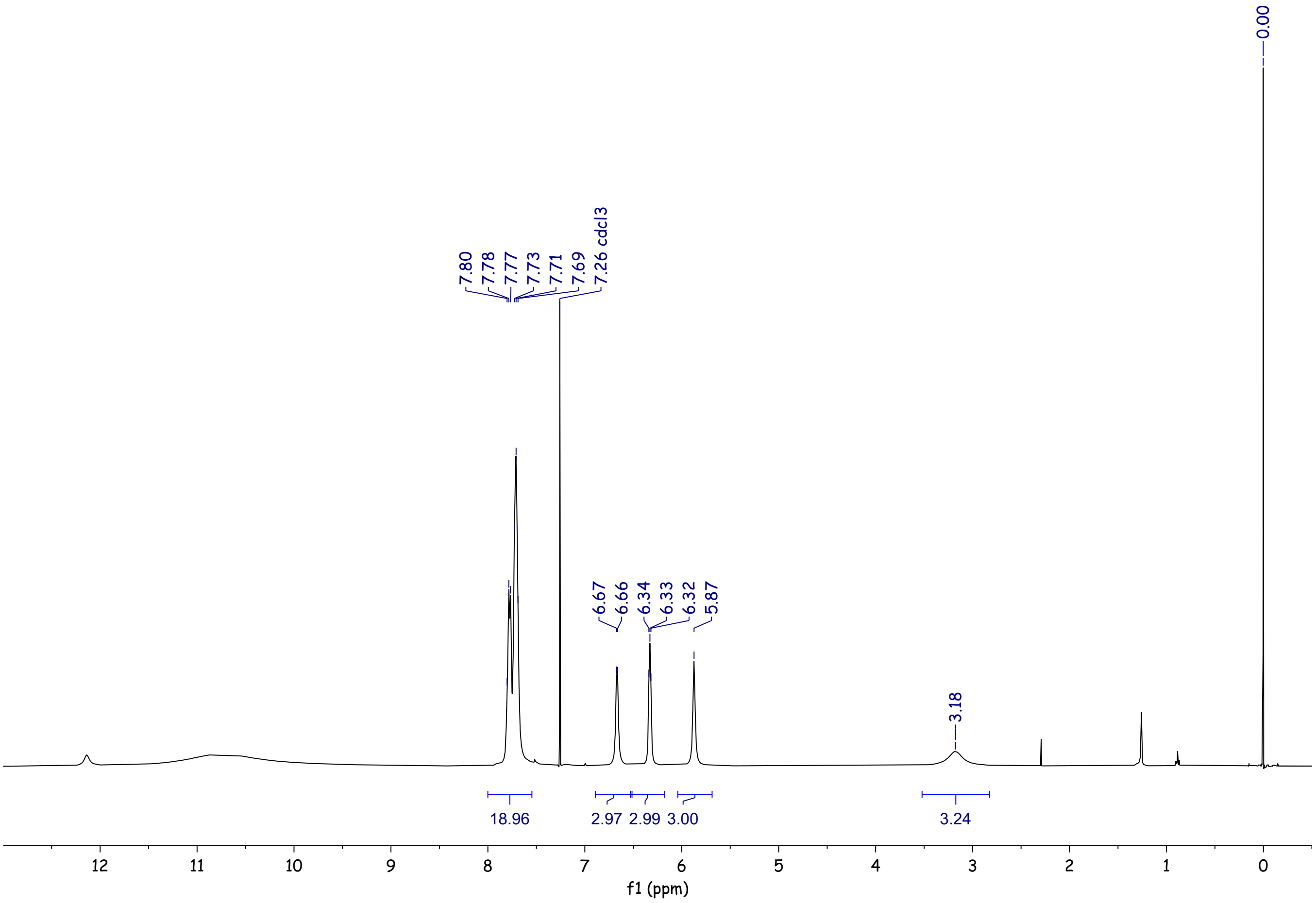

Figure S1f. ^{1}H-NMR spectra of the complex $[Eu(hth)_3(tppo)_2]$ (**5**) in $CDCl_3$.

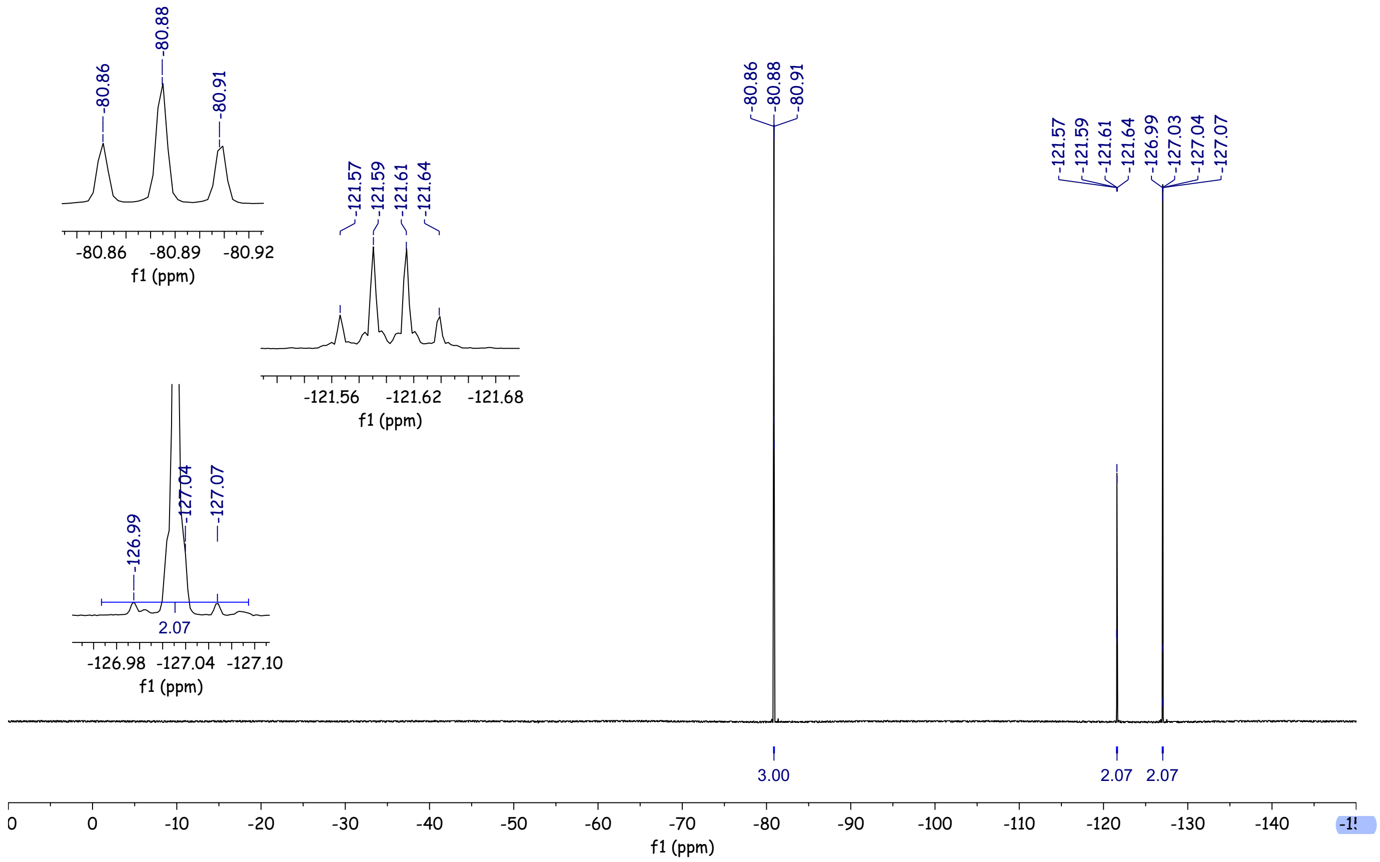

Figure S2a. ^{19}F-NMR spectra of the ligand **hth⁻Na⁺** in $CDCl_3$.

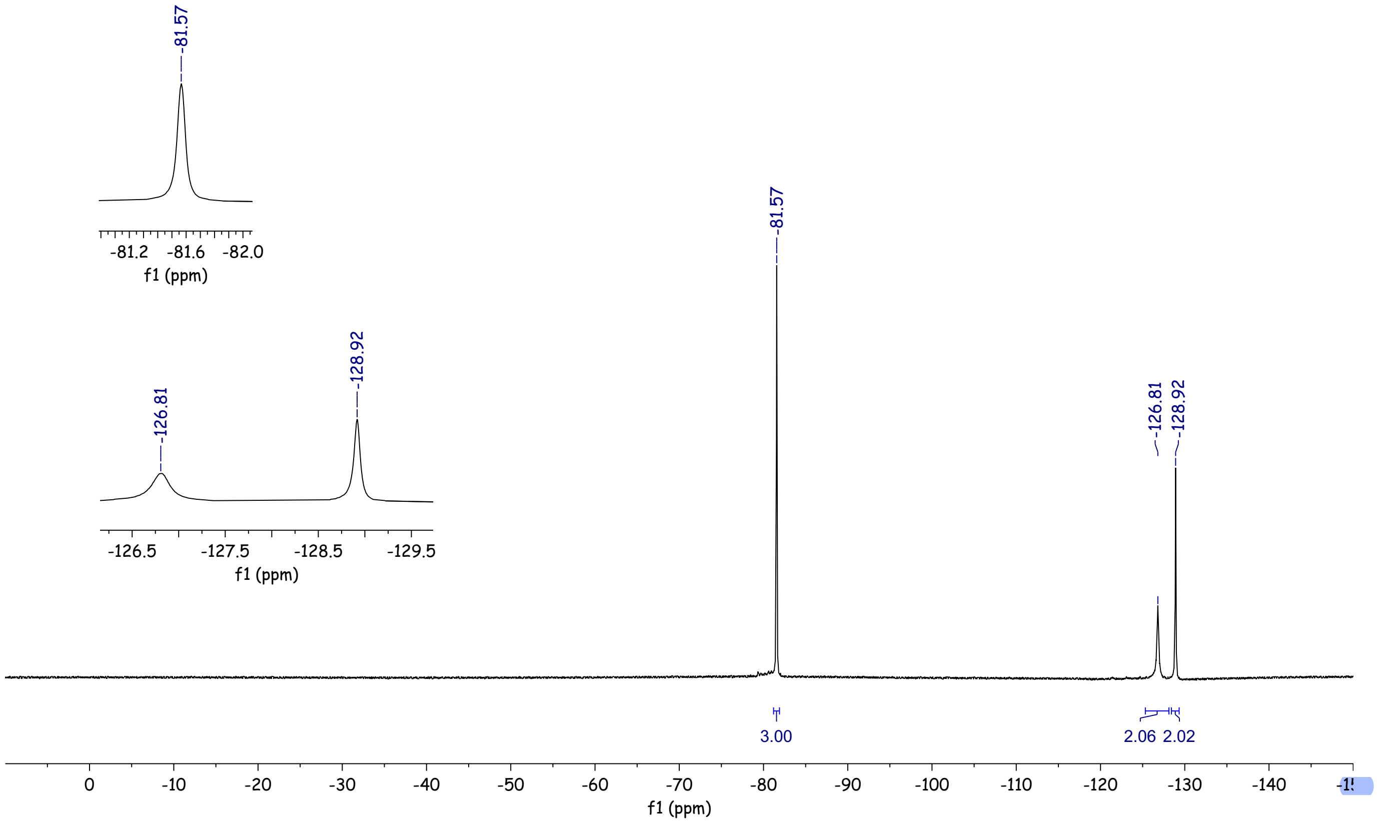

Figure S2b. ^{19}F-NMR spectra of the complex $[Eu(hth)_3(H_2O)_2]$ (**1**) in $CDCl_3$.

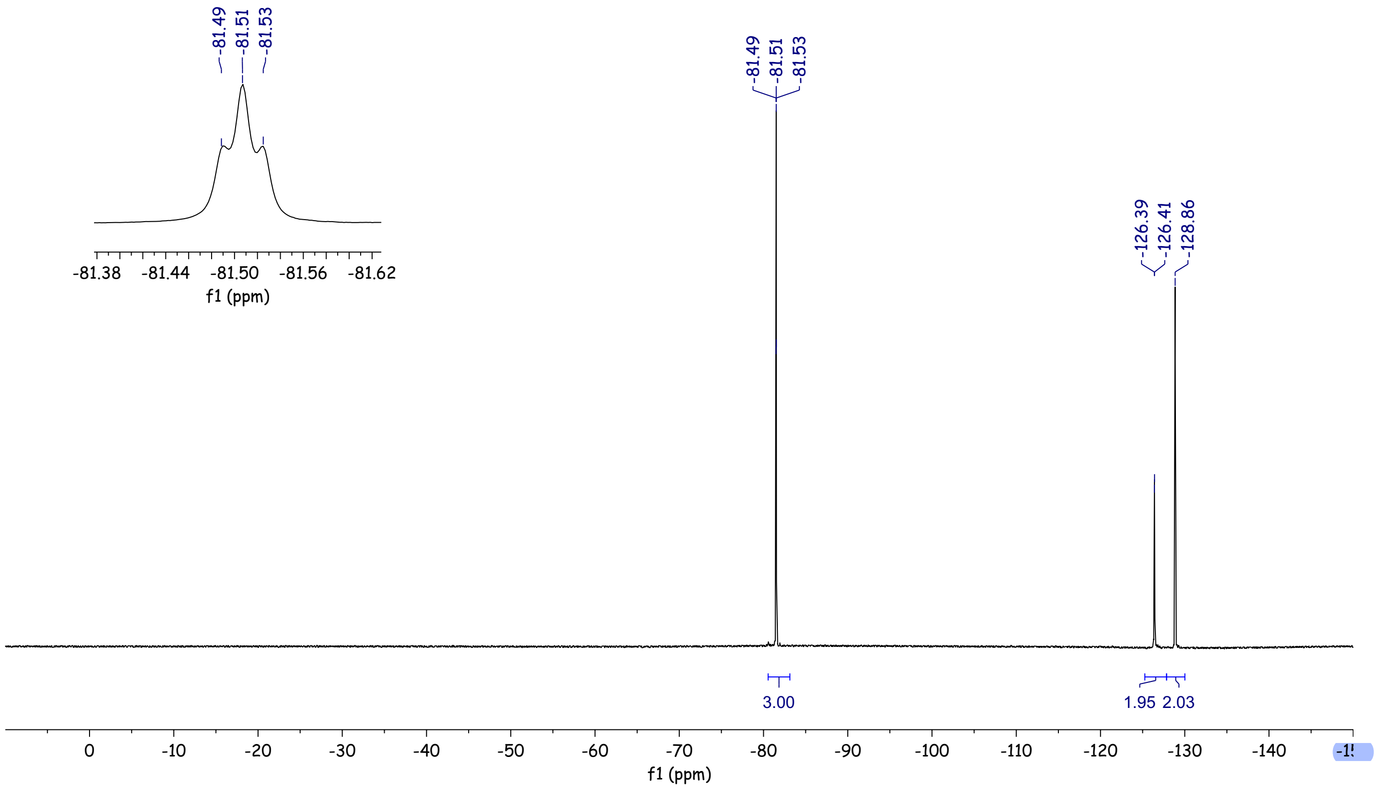

Figure S2c. ^{19}F-NMR spectra of the complex [Eu(hth)$_3$(dpso)$_2$] (**2**) in $CDCl_3$.

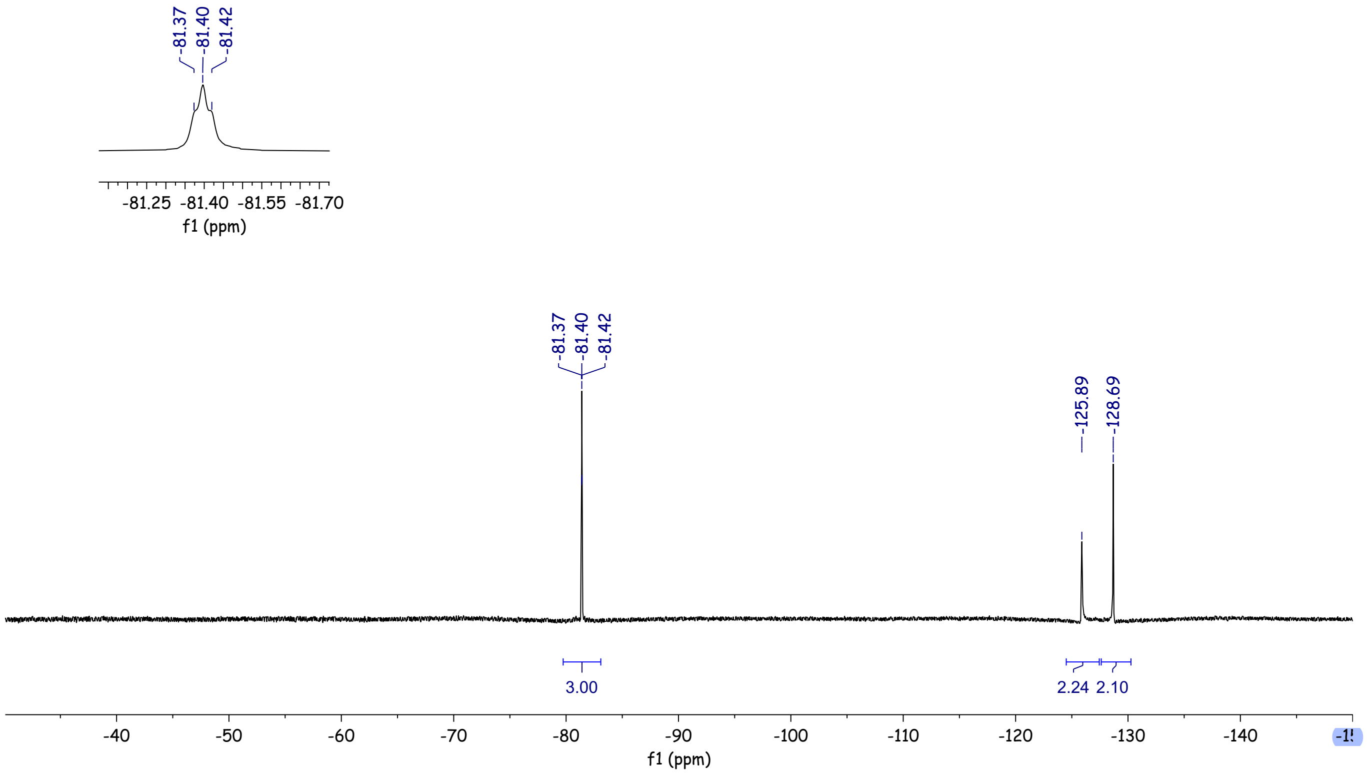

Figure S2d. ^{19}F-NMR spectra of the complex [Eu(hth)$_3$(dpsoCH$_3$)$_2$] (**3**) in $CDCl_3$.

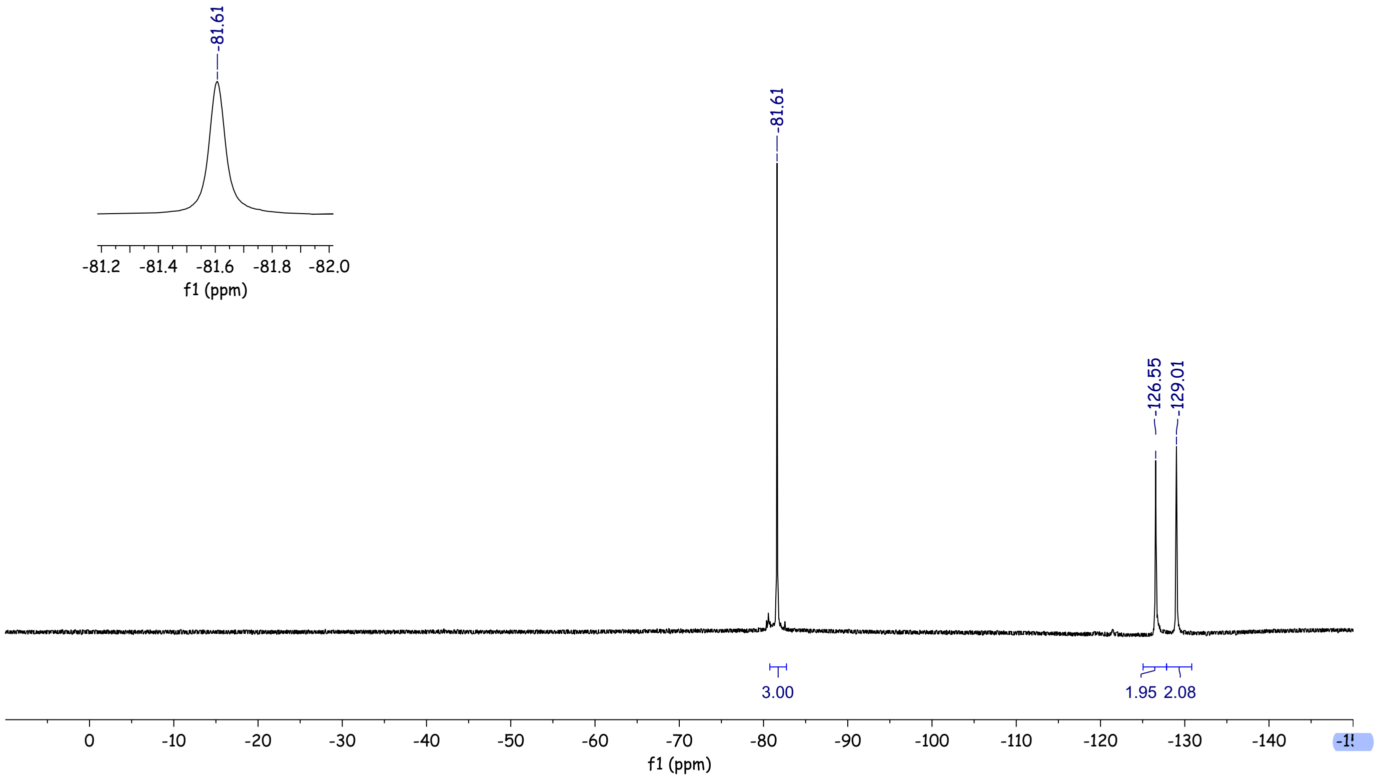

Figure S2e. ^{19}F-NMR spectra of the complex [Eu(hth)$_3$(dpsoCl)$_2$] (**4**) in $CDCl_3$.

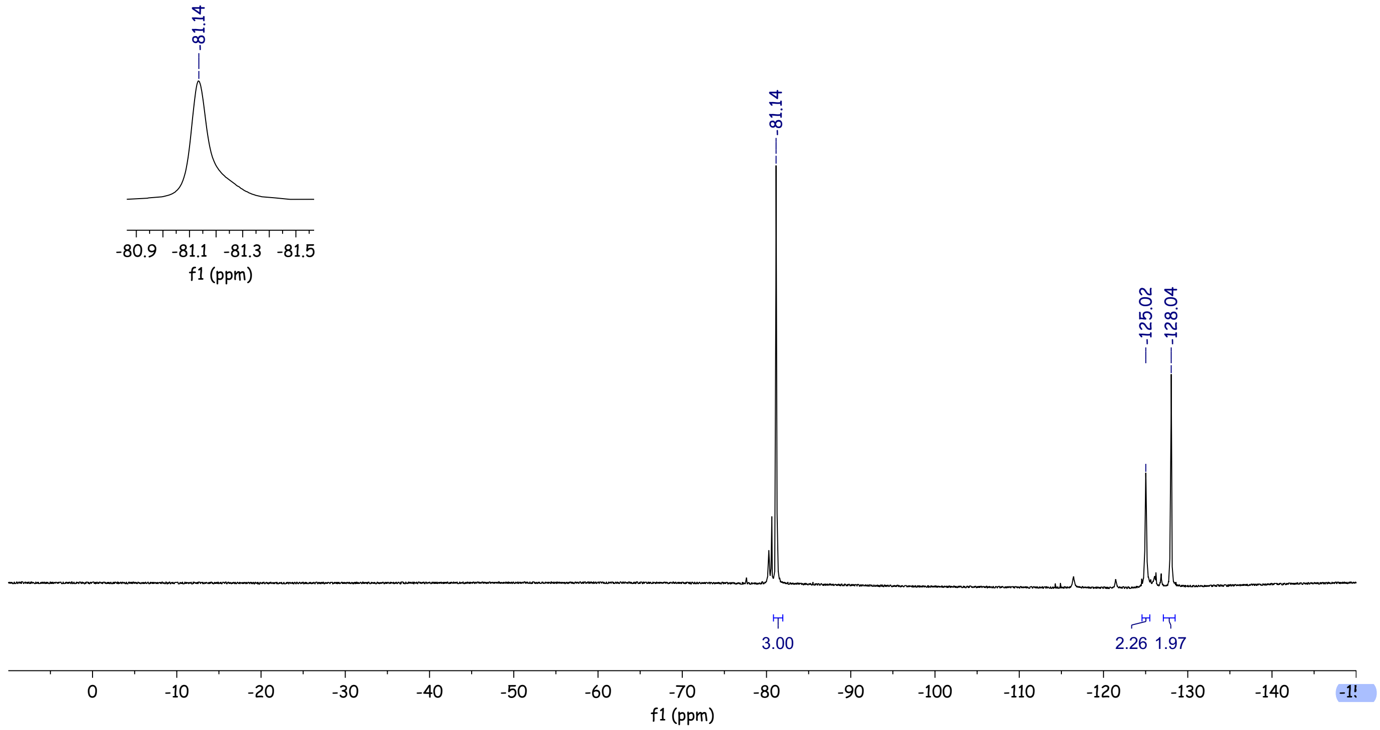

Figure S2f. ^{19}F-NMR spectra of the complex [Eu(hth)$_3$(tppo)$_2$] (**5**) in $CDCl_3$.

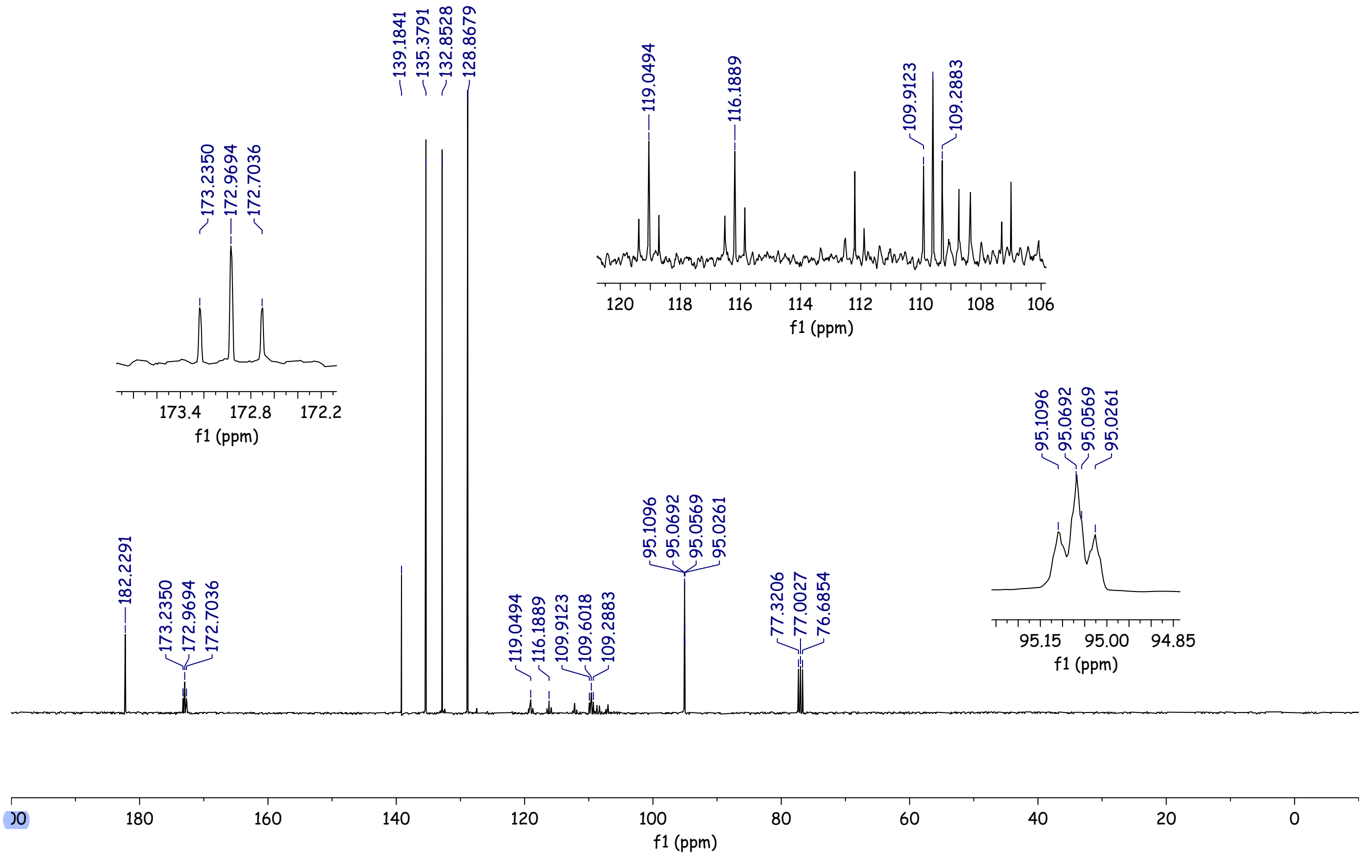

Figure S3a. ^{13}C-NMR spectra of the ligand **hth⁻Na⁺** in $CDCl_3$.

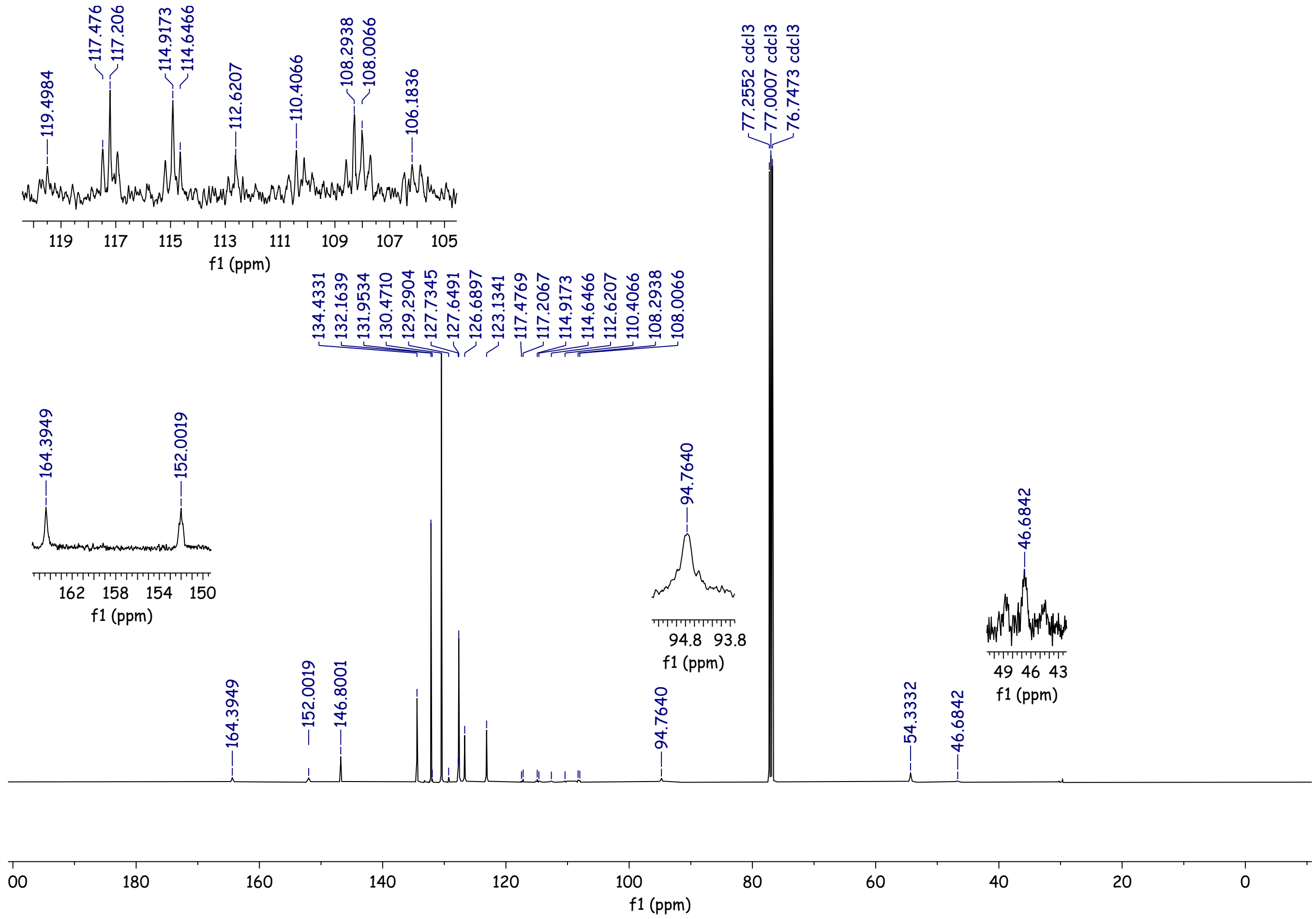

Figure S3b. ^{13}C-NMR spectra of the complex [Eu(hth)$_3$(dpso)$_2$] (**2**) in $CDCl_3$.

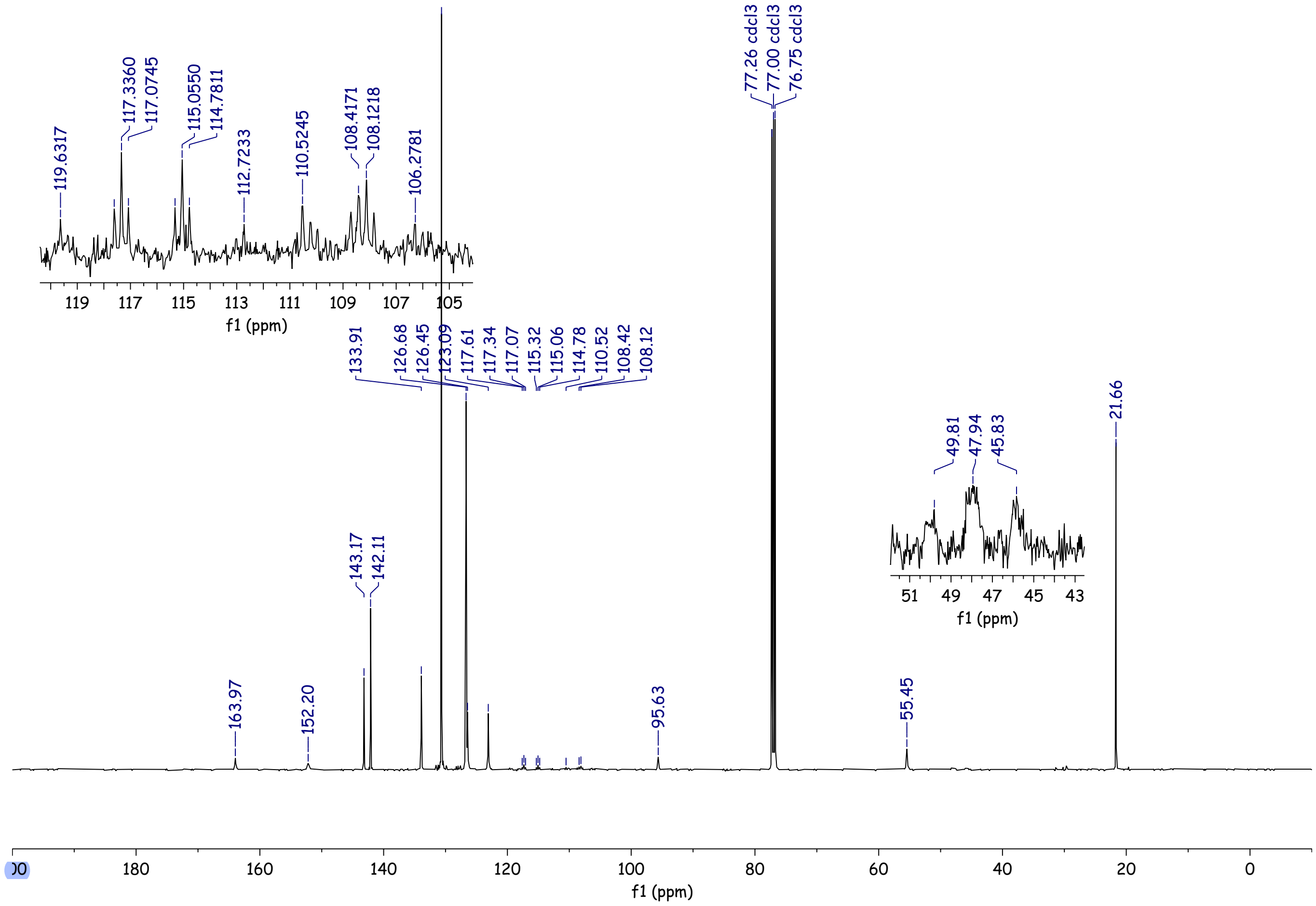

Figure S3c. ^{13}C-NMR spectra of the complex [Eu(hth)$_3$(dpsoCH$_3$)$_2$] (**3**) in $CDCl_3$.

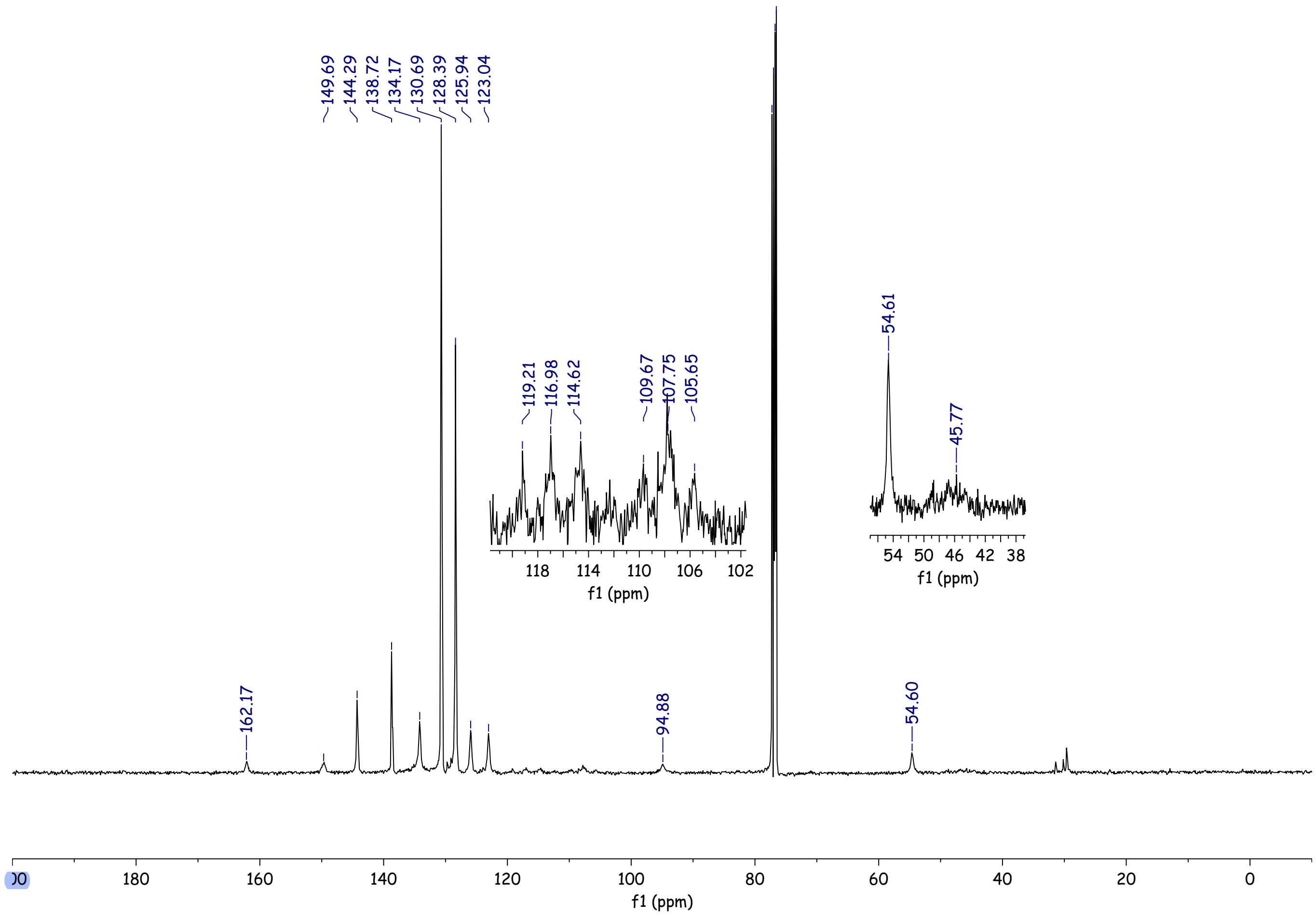

Figure S3d. ^{13}C-NMR spectra of the complex [Eu(hth)$_3$(dpsoCl)$_2$] (**4**) in $CDCl_3$.

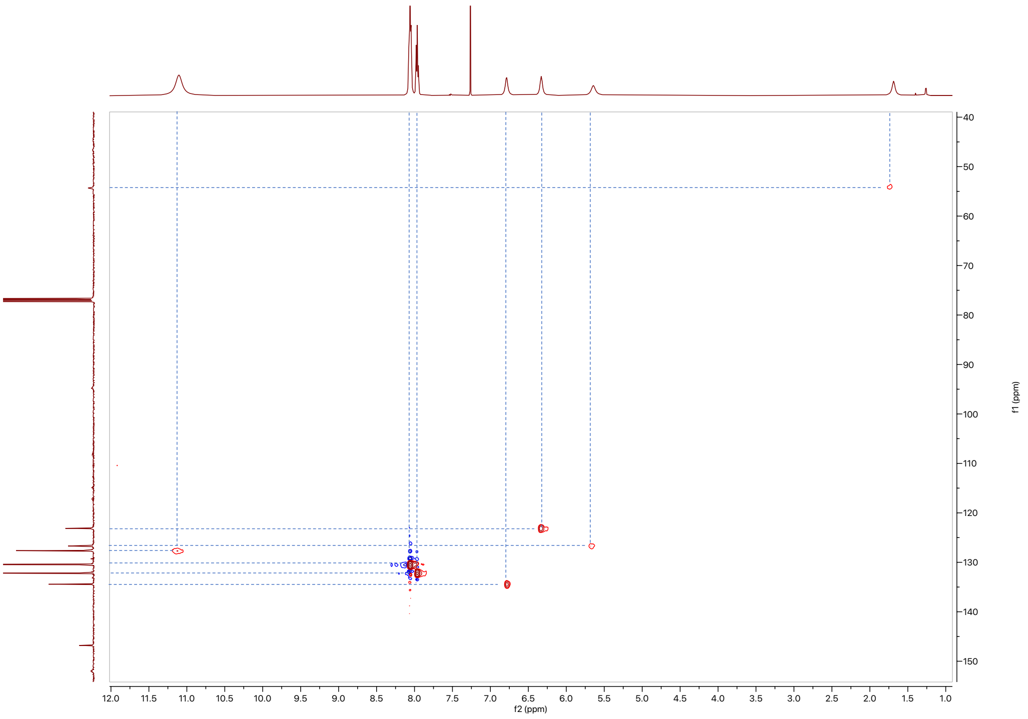

Figure S4a. HSQC NMR spectra of the complex [Eu(hth)$_3$(dpso)$_2$] (**2**) in $CDCl_3$.

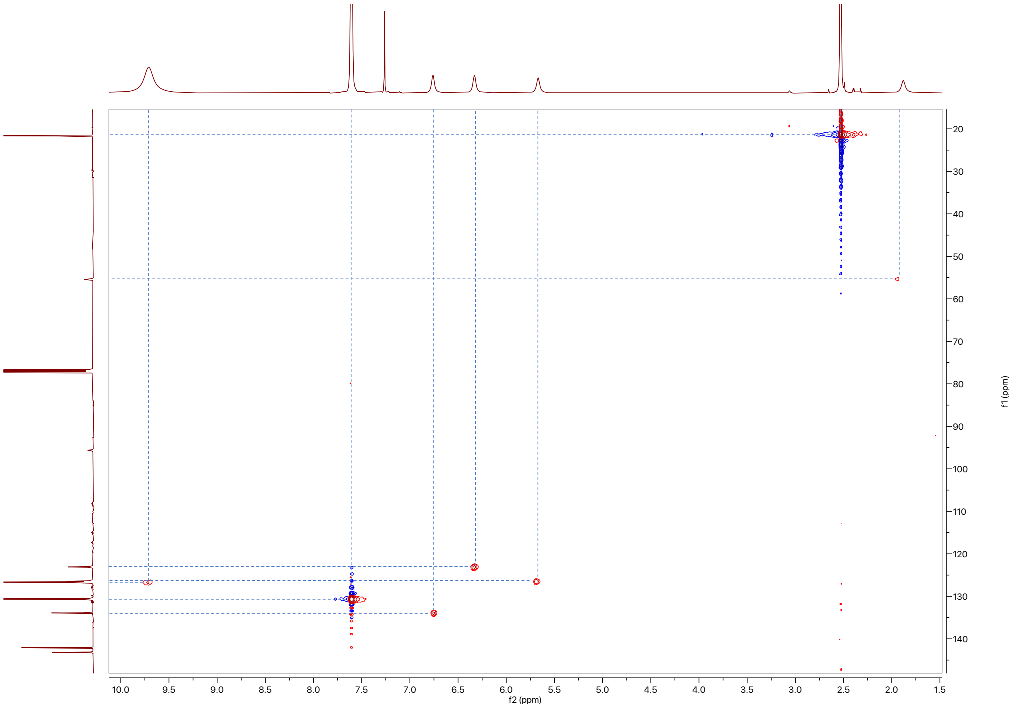

Figure S4b. HSQC NMR spectra of the complex [Eu(hth)$_3$(dpsoCH$_3$)$_2$] (**3**) in $CDCl_3$.

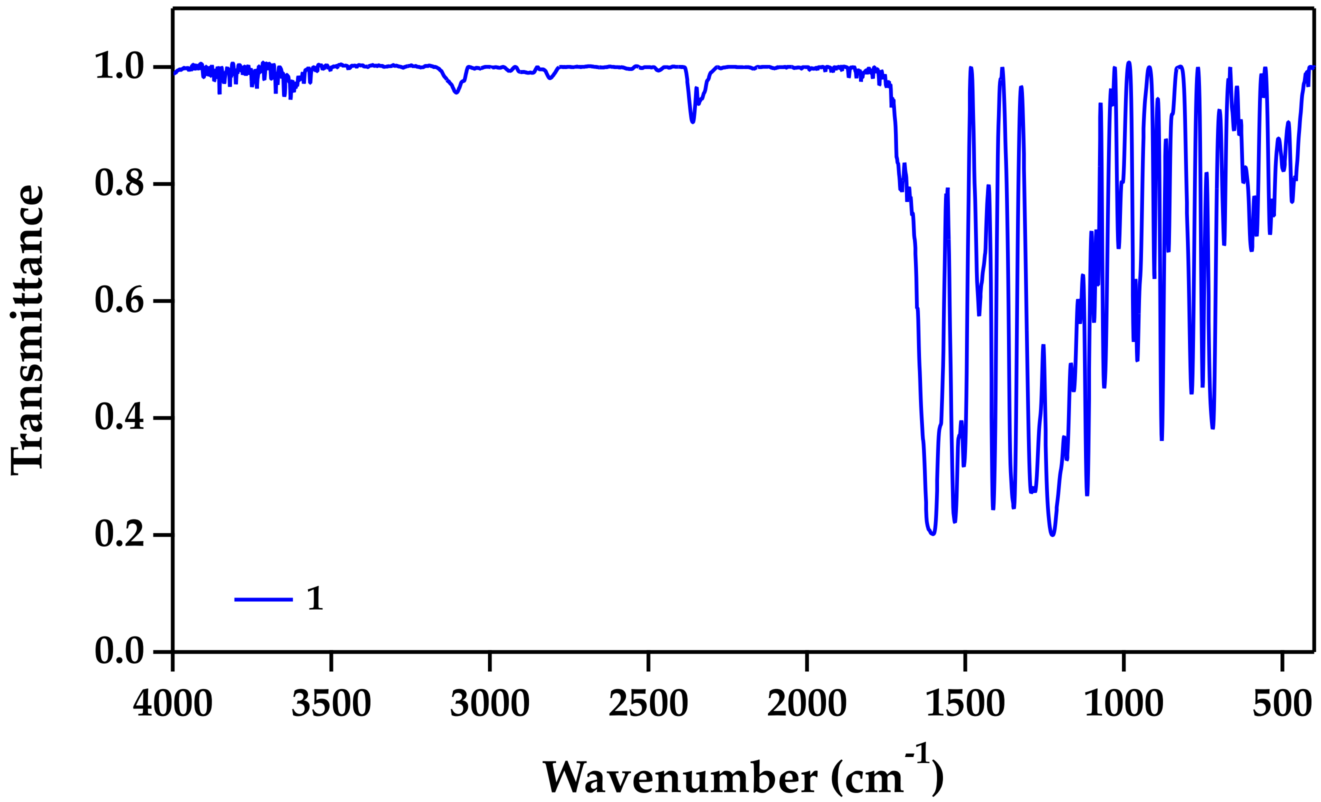

Figure S5a. Vibrational spectrum of the complex [Eu(hth)$_3$(H_2O)$_2$] (**1**).

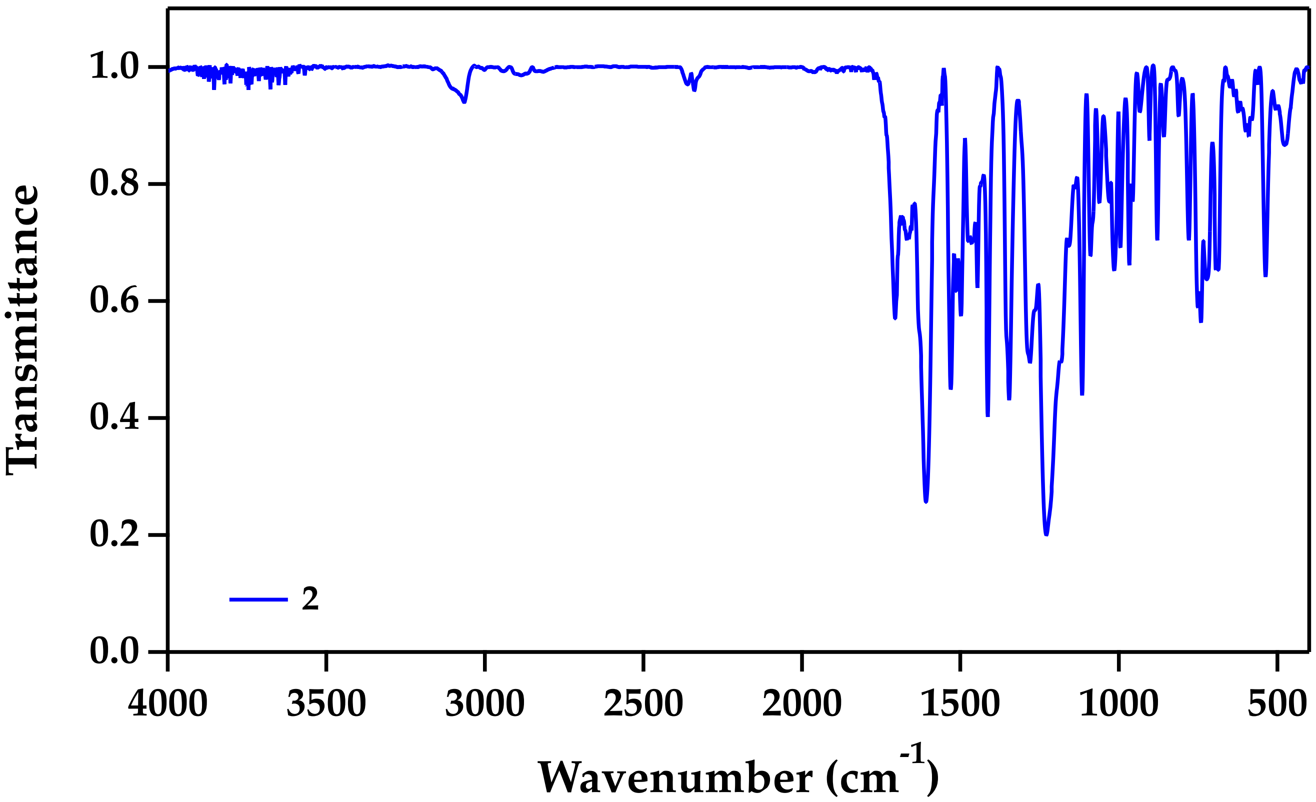

Figure S5b. Vibrational spectrum of the complex [Eu(hth)$_3$(dpso)$_2$] (**2**).

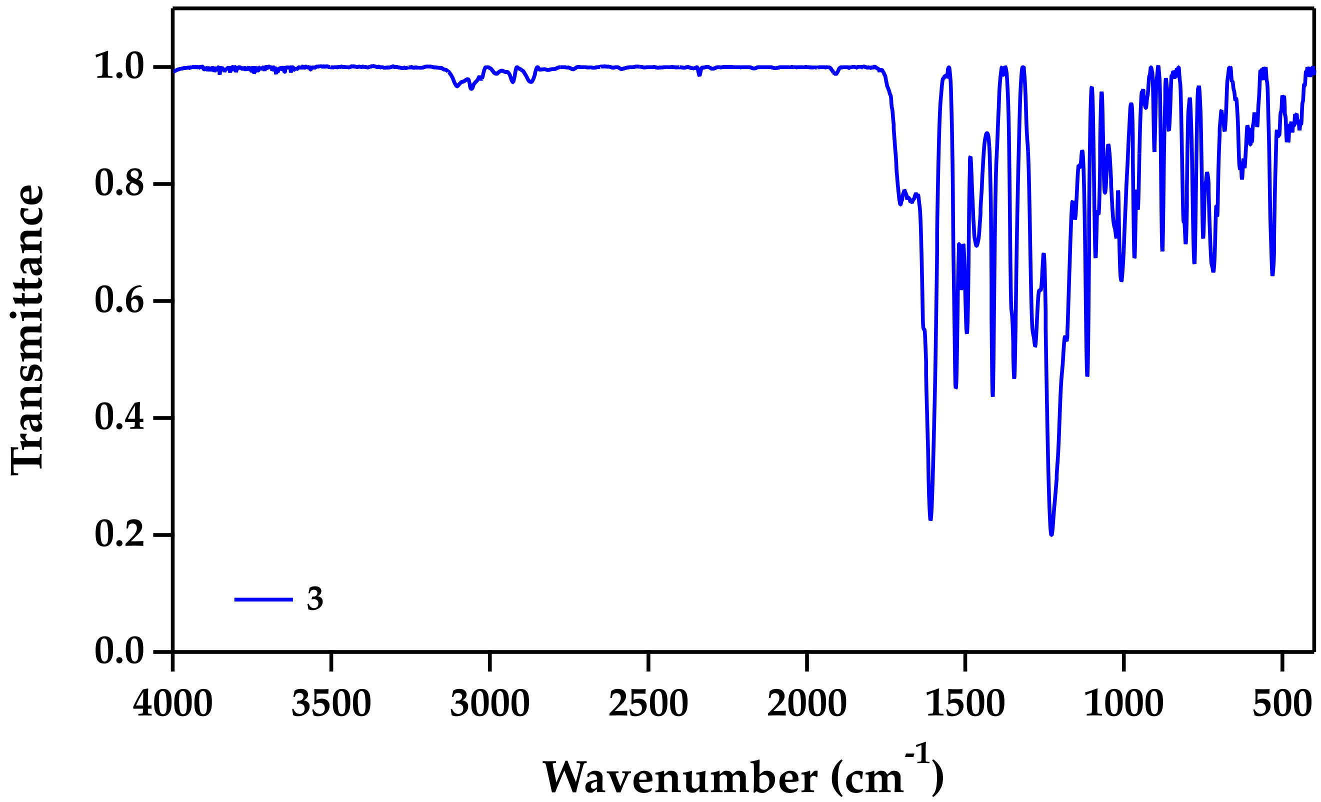

Figure S5c. Vibrational spectrum of the complex [Eu(hth)$_3$(dpsoCH$_3$)$_2$] (**3**).

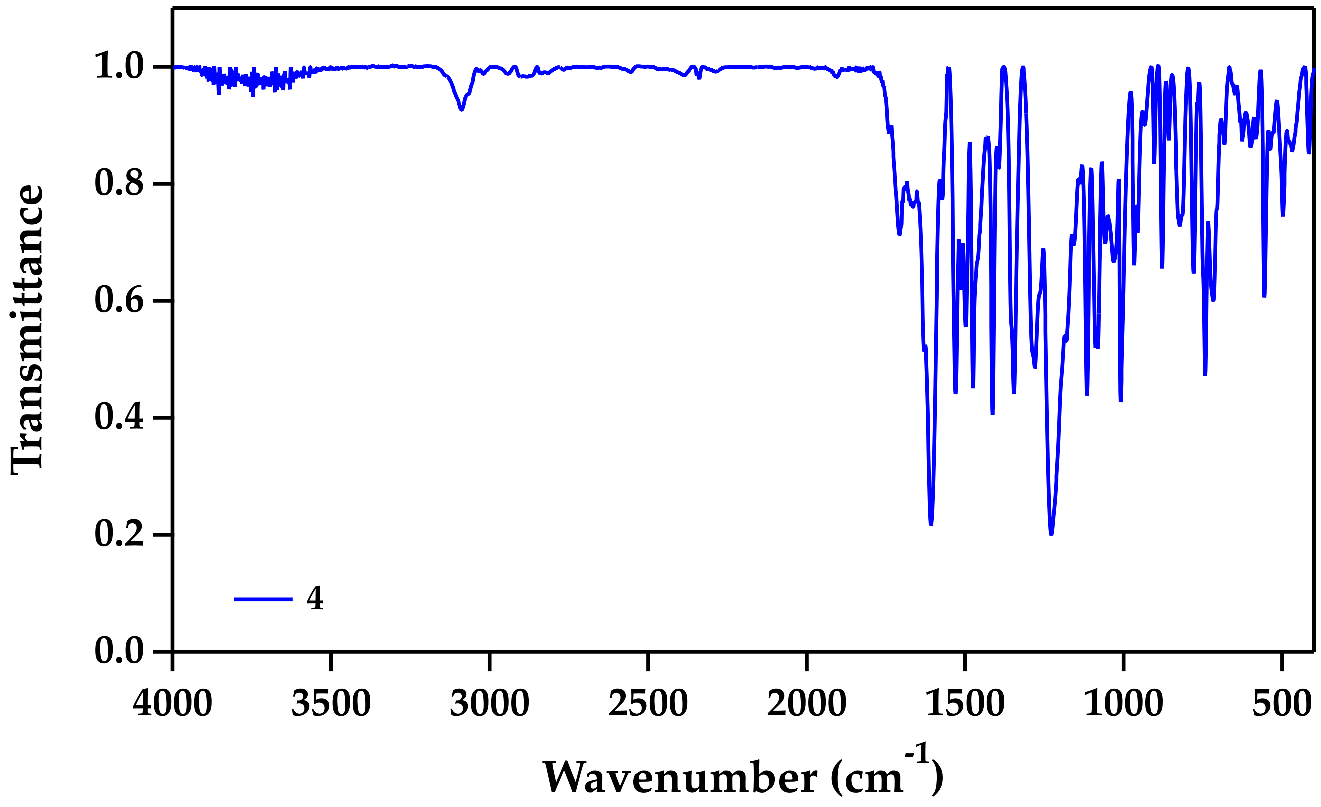

Figure S5d. Vibrational spectrum of the complex [Eu(hth)$_3$(dpsoCl)$_2$] (**4**).

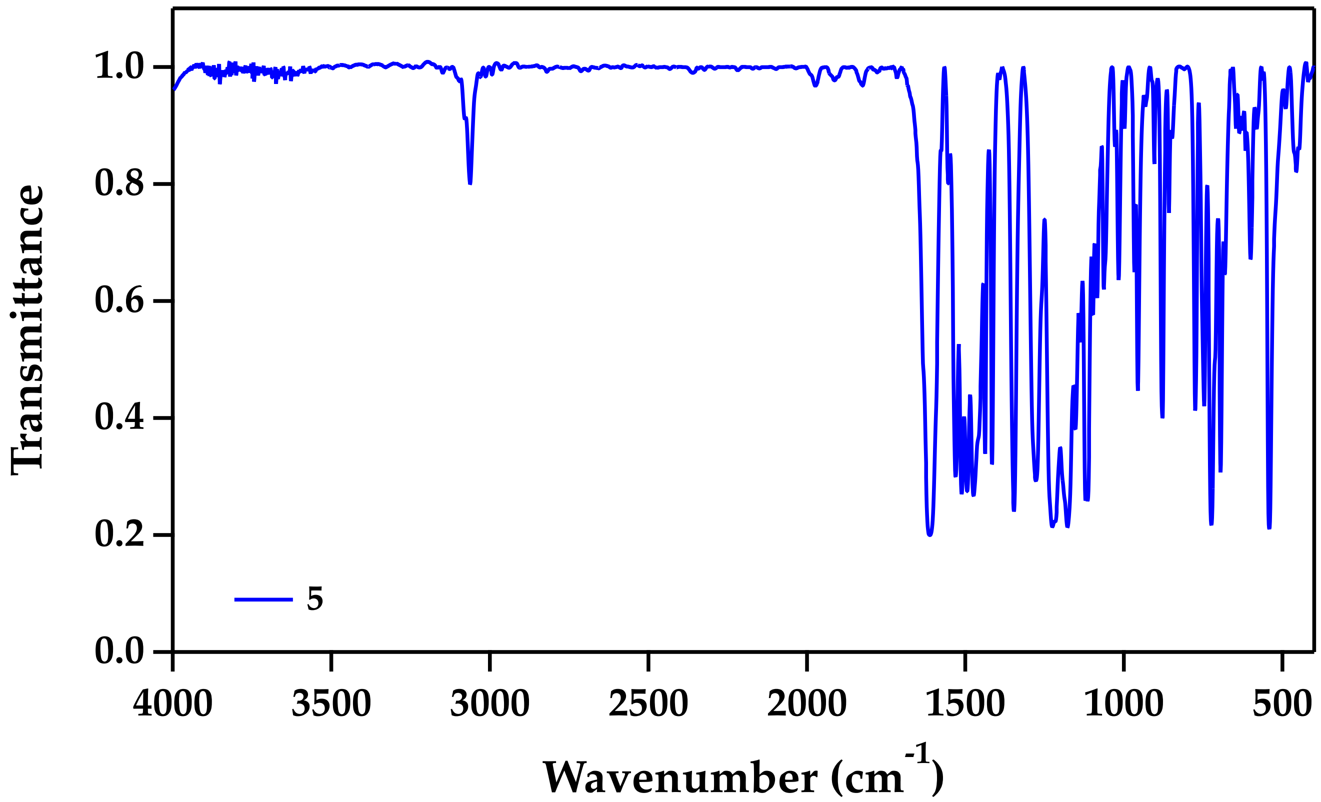

Figure S5e. Vibrational spectrum of the complex [Eu(hth)$_3$(tppo)$_2$] (**5**).

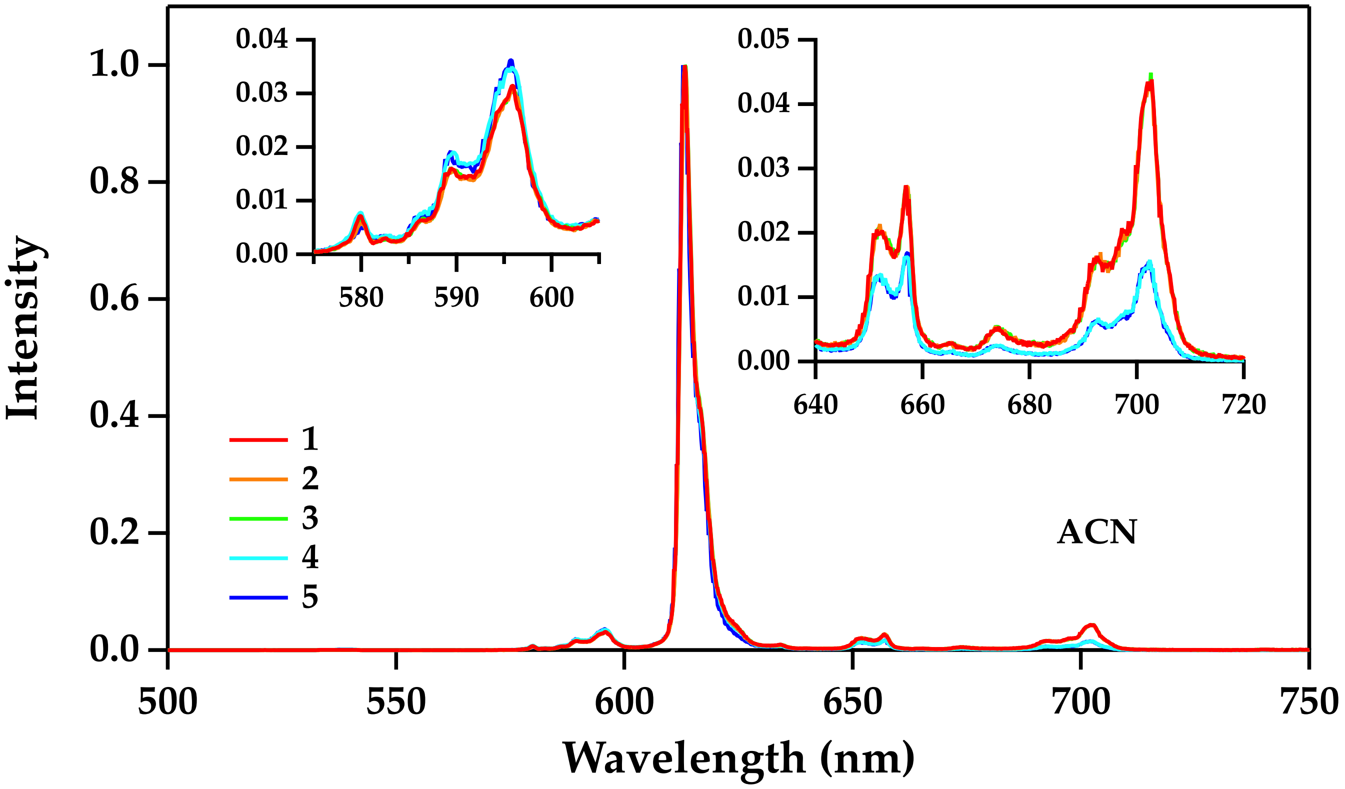

Figure S6a. Normalized emission spectra of the complexes **1-5** in acetonitrile solution at rt.

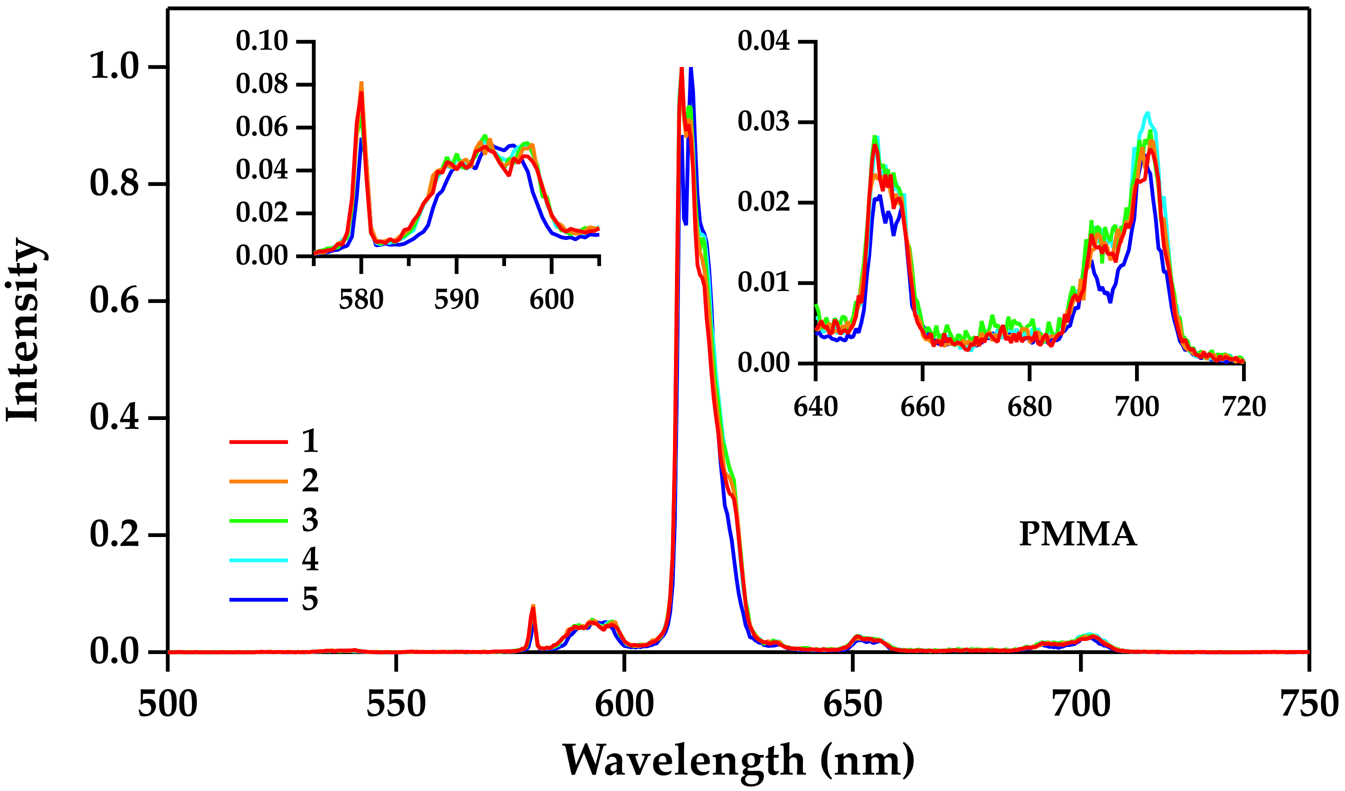

Figure S6b. Normalized emission spectra of the complexes **1-5** in PMMA film at rt.

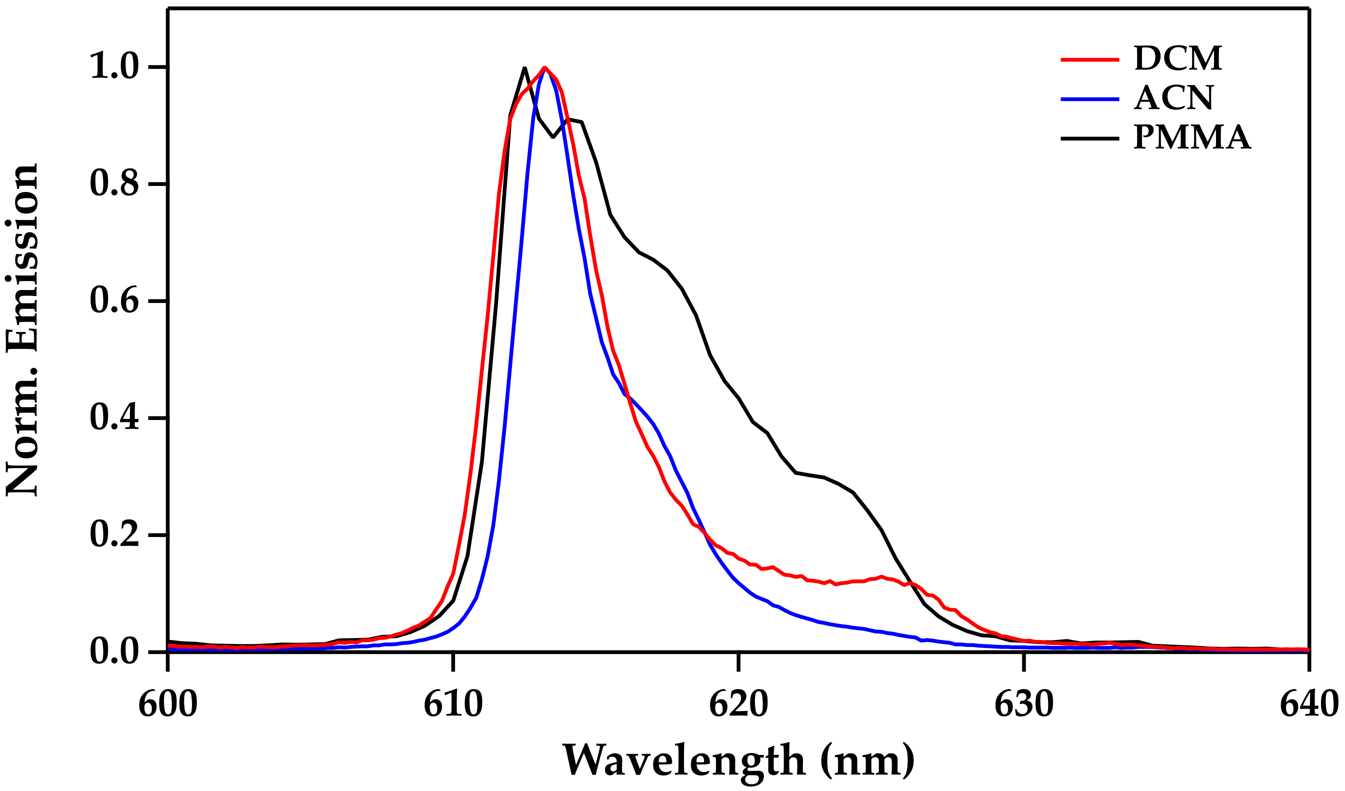

Figure S7. Band shapes of the $^5D_0 \rightarrow {}^7F_2$ transition of complex **2** in CH_2Cl_2, CH_3CN solutions and PMMA film.

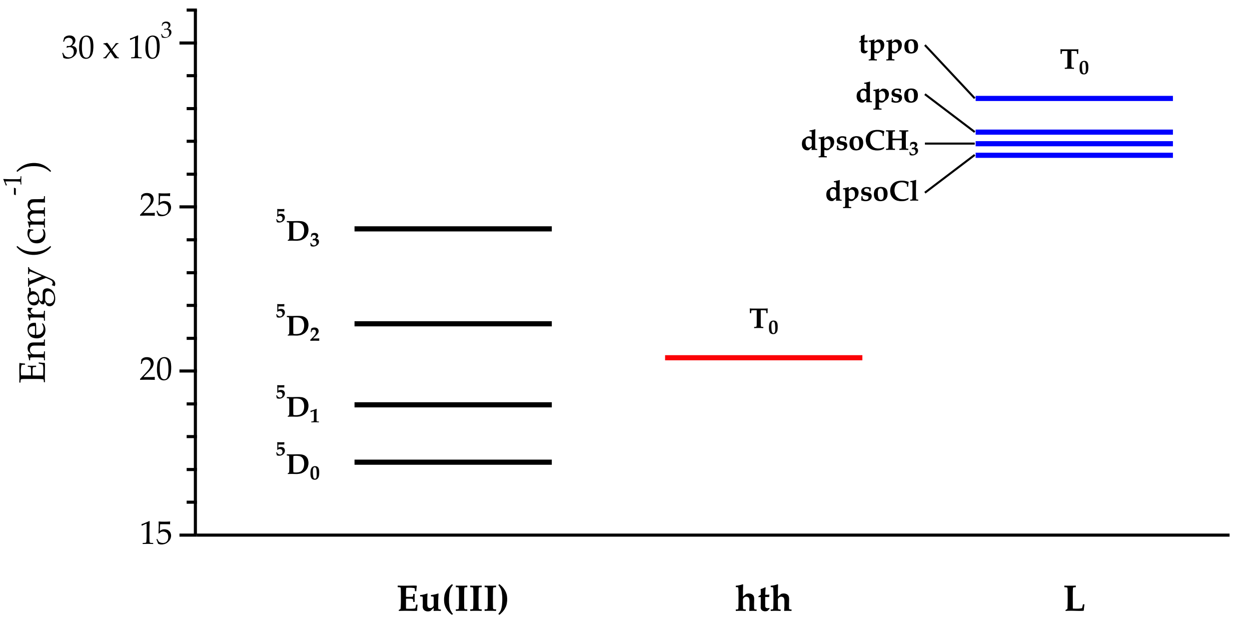

Figure S8. Energy levels diagram. The data for the ^5D_n excited states of Eu(III) are from ref [1], the triplet level of the β-diketonate ligand hth is from ref [2], and the triplet levels of co-ligands dpso, dpsoCH$_3$, dpsoCl are from ref [3] and tppo from ref [4].

1. Binnemans, K. Interpretation of europium(III) spectra. *Coord. Chem. Rev.* **2015**, *295*, 1-45.
2. Zheng, Y.; Fu, L.; Zhou, Y.; Yu, J.; Yu, Y.; Wang, S.; Zhang, H. Electroluminescence based on a β-diketonate ternary samarium complex. *J. Mater. Chem.* **2002**, *12*, 919-923.
3. Jenks, W.S.; Lee, W.; Shutters, D. Photochemistry and Photophysics of Aromatic Sulfoxides. 1 Characterization of the Triplets at Cryogenic Temperatures. *J. Phys. Chem.* **2002**, *98*, 2282-2289.
4. Li, H.; Hong, M.; Scarpaci, A.; He, X.; Risko, C.; Sears, J.S.; Barlow, S.; Winget, P.; Marder, S.R.; Kim, D.; et al. Chemical Stabilities of the Lowest Triplet State in Aryl Sulfones and Aryl Phosphine Oxides Relevant to OLED Applications. *Chem. Mater.* **2019**, *31*, 1507-1519.

Table S1. Crystal data and structure refinement of the complex **5**, the calculations were performed on the disordered $[Eu(hth)_3(tppo)_2]$ which contains the O1A and O1B.

Empirical formula	$C_{66}H_{42}EuF_{21}O_8P_2S_3$
Formula weight	1672.07
Temperature	293(2) K
Crystal system	Monoclinic
Space group	C 2/c
a	25.3565(11) Å
b	14.5812(4) Å
c	21.2962(9) Å
α	90°
β	116.653(5)°
γ	90°
Volume	7037.1(5)Å3
Z	4
Density (calculated)	1.578 Mg/m3
Absorption coefficient	1.134 mm^{-1}
F(000)	3328
Crystal size	0.43 x 0.18 x 0.11 mm^3
Radiation	MoKα (λ = 0.71073)
2Θ range for data collection/°	3.237 to 29.221°
Index ranges	$-29 \le h \le 34, -19 \le k \le 19, -28 \le l \le 21$
Reflections collected	31978
Independent reflections	8435 [R(int) = 0.0355]
Data / restraints / parameters	8435 / 32 / 557
Goodness-of-fit on F^2	1.283
Final R indexes [$I \ge 2\sigma$ (I)]	R1 = 0.0804, wR2 = 0.1754
Final R indexes [all data]	R1 = 0.0973, wR2 = 0.1823
Largest diff. peak/hole	0.803 and –0.784 e·$Å^{-3}$

Table S2. Selected bond lengths (Å) of the complex **5**.

O1A – Eu1	2.377 (13)
O1B – Eu1	2.484 (14)
O2 – Eu1	2.393 (4)
O3 – Eu1	2.350 (4)
O4 – Eu1	2.383 (5)

Table S3. Sensitization efficiencies.

	ϕ, %	τ, μs	η_{sens}, %
**1**	55.7	574	93
**2**	59.4	584	98
**3**	51.3	576	86
**4**	57.5	583	95
**5**	65.8	597	106

The sensitization efficiency, η_{sens}, has been calculated from the equation:

$$\phi = \eta_{sens} \frac{\tau}{\tau_{rad}}$$

where τ_{rad} is the radiative lifetime calculated from the equation:

$$\frac{1}{\tau_{rad}} = A_{MD,0}\, n^3 \left(\frac{I_{tot}}{I_{MD}}\right)$$

For the details of the calculation see Binnemans, K. Interpretation of europium(III) spectra. *Coord. Chem. Rev.* **2015**, *295*, 1-45.
